# An explainable imaging-clinical biomarker for non-small cell lung cancer prognostication based on normalised hotspot to centroid distance and [^18^F]FDG PET/CT radiomics

**DOI:** 10.1007/s00259-025-07659-4

**Published:** 2025-12-12

**Authors:** Mitchell Chen, Susan J. Copley, Yidong Han, Mubarik A. Arshad, Patrizia Viola, Kristofer Linton-Reid, Tina Stoycheva, Gary J. R. Cook, David Landau, Sue Chua, Richard O’Connor, Jeannette Dickson, Danielle Power, Andrea G. Rockall, Tara D. Barwick, Eric O. Aboagye

**Affiliations:** 1 Department of Surgery and Cancer, Imperial College London, Hammersmith Hospital Campus, Du Cane Road, London, W12 0NN UK; 2 Department of Radiology & Nuclear Medicine, Imperial College Healthcare NHS Trust, Hammersmith Hospital, Du Cane Road, London, W12 0HS UK; 3 Institute of Nuclear Medicine, University College London, London, NW1 2BU UK; 4 North West London Pathology, Charing Cross Hospital, London, W6 8RF UK; 5 Department of Cancer Imaging, School of Biomedical Engineering and Imaging Sciences, King’s College London, St. Thomas’ Hospital, Westminster Bridge Road, London, SE1 7EH UK; 6 Department of Nuclear Medicine, The Royal Marsden Hospital, Downs Road, Sutton, SM2 5PT UK; 7 Department of Nuclear Medicine, Queen’s Medical Centre, Derby Road, Nottingham, NG7 2UH UK; 8 Department of Clinical Oncology, Mount Vernon Hospital, Rickmansworth Road, Northwood, HA6 2RN UK; 9 Department of Clinical Oncology, Imperial College Healthcare NHS Trust, Hammersmith Hospital, Du Cane Road, London, W12 0HS UK

**Keywords:** Radiomics, [^18^F]FDG, PET/CT, NSCLC, Prognosis, Explainable AI

## Abstract

**Purpose:**

Accurate prognostication is crucial for guiding personalised treatment strategies in non-small cell lung cancer (NSCLC). While radiomics offers promise, few features are derived from cancer models with causal justification to support their biological validity. This study evaluated the prognostic utility of normalised hotspot-to-centroid distance (NHOC), a recently proposed [18F]FDG PET imaging metric derived from a cancer evolutionary model, and its integration with PET/CT radiomics and clinical features to form a composite signature, non-invasive lung cancer evolution vector (nLCEV).

**Methods:**

A retrospective, multi-centre study was conducted using pre-treatment [18F]FDG PET/CT scans from 285 NSCLC patients (mean age: 67.7 ± 10.1 years; male:female = 171:114, International Association for the Study of Lung Cancer stage: T1/2/3/4/unknown = 61/118/53/52/1, N0/1/2/3/unknown = 133/46/71/34/1, M0/1/unknown = 222/62/1) from Imperial College Healthcare NHS Trust as the discovery cohort. External validation cohorts included patients from King’s College (*n* = 53), Royal Marsden (*n* = 63), Mount Vernon (*n* = 61), and Nottingham University (*n* = 38) hospitals. NHOC was evaluated for 3-year overall survival prediction and combined with a multi-regional PET/CT radiomics predictive vector (RPV) and disease stage to develop nLCEV.

**Results:**

NHOC and RPV demonstrated independent prognostic value (hazard ratio (HR) [95% confidence interval]: 2.52 [1.60–3.98] and 2.68 [2.13–3.38], respectively). nLCEV achieved an area under the receiver operating characteristic curve of 0.76 [0.60–0.92] and stratified patients into high- and low-risk groups across all validation cohorts with significant HR: KCL 3.27 [1.31, 8.16], Marsden 2.21 [1.02, 4.78], Mount Vernon 2.60 [1.42, 4.76], and Nottingham 4.14 [1.44, 11.90] (all p < 0.05).

**Conclusion:**

NHOC enhances NSCLC patient survival prediction, and when integrated with PET-CT radiomics and disease stage, offers a robust, non-invasive approach to disease prognostication.

**Supplementary Information:**

The online version contains supplementary material available at 10.1007/s00259-025-07659-4.

## Introduction

Lung cancer is the leading cause of cancer-related deaths worldwide, with non-small cell lung cancer (NSCLC) accounting for 80–85% of its cases [1]. Over 70% of NSCLC cases are diagnosed at advanced stages, carrying poor prognoses [2]. Disease prognostication is critically important for guiding personalised treatment decisions, improving clinical outcomes and enhancing patients’ understanding of their disease trajectory.

Radiomic features are quantitative metrics derived from imaging data and can non-invasively capture important disease information⁠ [3, 4]. As part of their disease work-up, NSCLC patients routinely receive ^18^F-fluorodeoxyglucose positron emission tomography/computed tomography ([^18^F]FDG PET/CT), which offers a prime window of opportunity to harness the power of radiomics for patient prognostication. Prior studies have demonstrated the utility of [^18^F]FDG PET/CT radiomics to achieve prognostication [5–11], but few have based their approach on a comprehensible biophysical disease model established a priori [12–14]. Cancer biology-derived imaging metrics offer better explainability and causal backing unparalleled by traditional data mining-derived signatures.

**Fig. 1:**
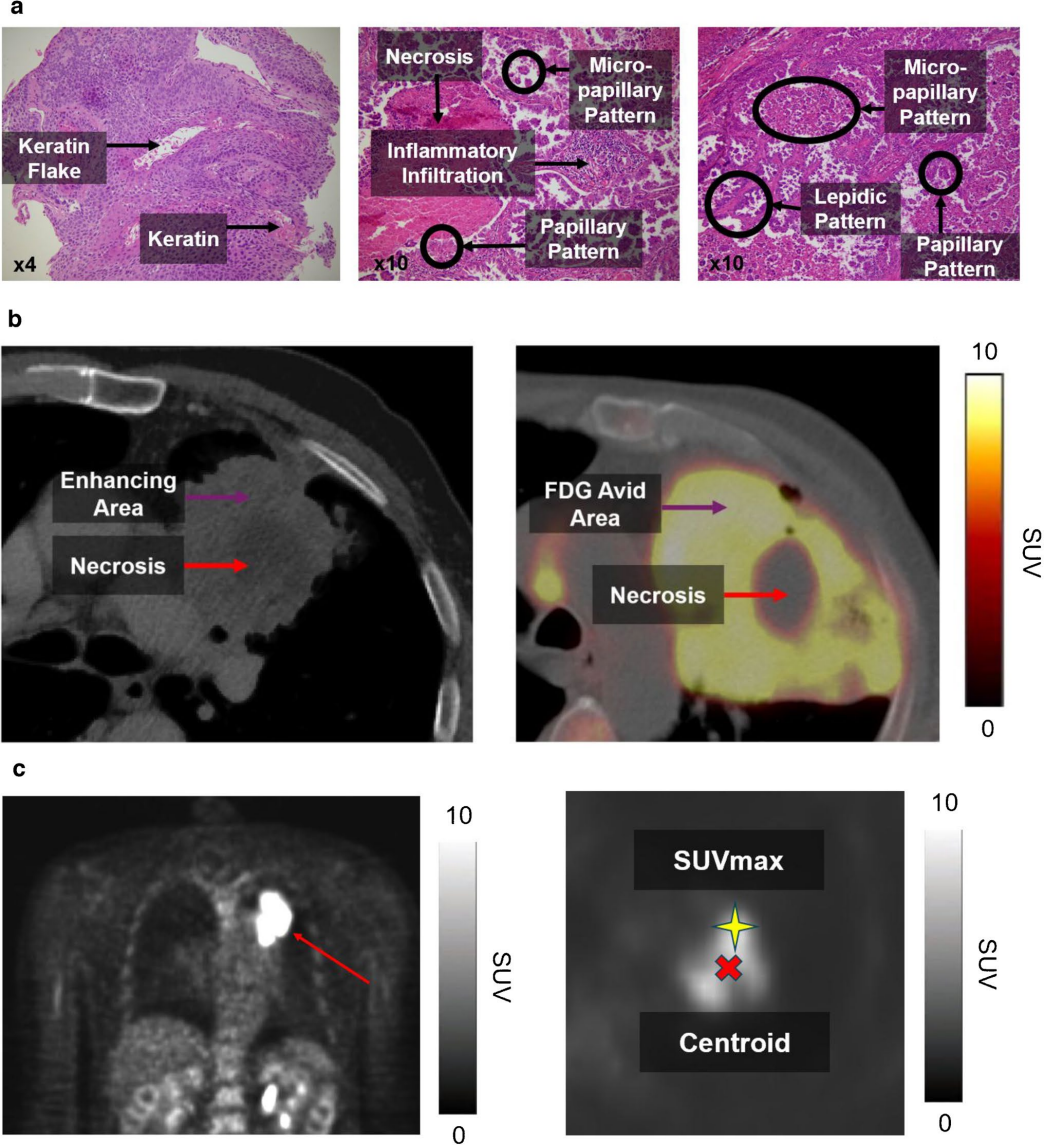
**a**. Intra-tumoural heterogeneity (ITH) appreciated histologically in a case of squamous cell carcinoma at 4 × magnification for (*left*) and two cases of adenocarcinoma at 10 × magnification (*middle* and *right*). **b**. ITH appreciated radiologically on CT (*left*) and [^18^F]FDG PET (*right*). **c**. Illustration of normalised hotspot to centroid (NHOC) distance, modelling postulated competition between cancer cell subpopulations

NSCLC demonstrates significant intra-tumoural heterogeneity [15]. Tumour-promoting driver cells show non-uniform spatial distribution. On contrast-enhanced CT, they can manifest as areas of increased enhancement or internal vascularity (Fig. 1a), reflecting their high perfusion/vascularity and/or angiogenesis favouring disease progression [16]. On [^18^F]FDG PET, such cells can exhibit higher avidity from their increased metabolic activity (Fig. 1b) due to Warburg effect, or an upregulation of glycolysis and glucose consumption in response to tumour microenvironment, hypoxia, and intrinsic oncogenic signalling [17]. As cancer evolves, areas of high metabolic activity are believed to drift towards the periphery as the tumour becomes more aggressive and are associated with poorer disease prognoses [18]. The normalised hotspot to centroid (NHOC) distance is a novel biomarker postulated to model this relationship, which is defined as the minimum Euclidean distance between the maximum standard uptake value ($SU{V}_{max}$) voxel and geometric centroid, normalised by the tumour’s metabolic spherical radius (MSR). It provides a dimensionless measure of the relative peripherality of the metabolic hotspot, such that the further the distance for a given tumour size, the more aggressive the cancer is anticipated to be. The prognostic value of NHOC has been tested *in silico* and in a small NSCLC dataset but was yet to be validated on at scale [18].

In this study, we sought to provide this validation in the context of NSCLC by evaluating its performance in several independent external cohorts and investigate its prognostic utility alongside conventional hand-crafted radiomics extracted from [^18^F]FDG PET and attenuation correction CT, to develop an effective composite non-invasive NSCLC evolutionary vector for patient prognostication.

## Material and methods

### Data collection

This retrospective study was approved by the institutional review board and Health Research Authority UK (HRA 18HH4616), conducted in accordance with the Declaration of Helsinki, and adhered to the STROBE and REMARK guidelines. The requirement for informed consent was waived due to the study’s retrospective and observational nature and use of de-identified patient data.

The inclusion criteria were patients with histologically confirmed NSCLC, with a target lesions ≥ 5 ml who had a pre-therapy [^18^F]FDG PET/CT scan available and underwent radical radiotherapy with or without chemotherapy. A minimum lesion volume of 5 ml was selected, based on early work [19]. Exclusion criteria were patients undergoing surgery, palliative treatment or disease showing low FDG avidity preventing reliable segmentation on [^18^F]FDG PET.

The discovery cohort consisted of pre-therapy [^18^F]FDG PET/CT scans from 285 NSCLC patients (age: 67.7 ± 10.1, male: female (M: F) = 171:114), acquired between July 2009 and November 2018 at Imperial College Healthcare NHS Trust (ICHNT). Disease staging was based on International Association for the Study of Lung Cancer (IASLC) 7th edition, applicable at the time of disease diagnosis: T1/2/3/4/unknown = 61/118/53/52/1, N0/1/2/3/unknown = 133/46/71/34/1, M0/1/unknown = 222/62/1. All patients were treated in line with the best available standard-of-care at the time of diagnosis, based on prevailing national and institutional guidelines.

The discovery cohort was split into training and internal validation sets of 232:53 (80:20 split), balanced for patient’s age, histology, stage and prognosis; in keeping with previous relevant literature [11, 14]. Independent external [^18^F]FDG PET/CT data were acquired between October 2008 and December 2013 at four other UK centres, also used in previous works [11, 20]: King’s College London & Guy’s and St. Thomas’ PET Centre, London (KCL: *n* = 53, age: 65.8 ± 9.2, M: F = 31:22, staging: T1/2/3/4/unknown = 6/14/15/17/1, N0/1/2/3/unknown = 10/5/33/4/1, M0/1/unknown = 41/11/1), Mount Vernon Hospital, Northwood (Mount Vernon: *n* = 61, age: 71.2 ± 9.5, M: F = 32:29, staging: T1/2/3/4/unknown = 22/10/25/1/3, N0/1/2/3/unknown = 22/10/25/1/3, M0/1/unknown = 60/0/1), Royal Marsden Hospital, Sutton (Marsden: *n* = 63, age: 68.1 ± 14.3, M: F = 43:20, staging: T1/2/3/4 = 11/17/17/18, N0/1/2/3 = 26/12/24/1, M0/1 = 62/1) and Nottingham University Hospital, Nottingham (Nottingham: *n* = 38, age: 69.7 ± 10.4, M: F = 21:17, staging: T1/2/3/4 = 3/14/19/2, N0/1/2/3 = 17/5/11/5, M0/1 = 37/1).

**Fig. 2:**
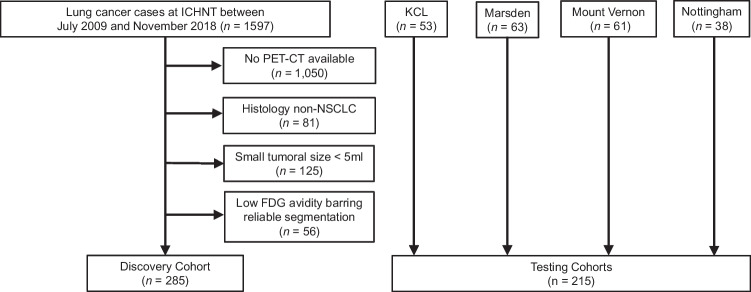
CONSORT diagram of the study cohorts. The external validation cohorts (KCL, Marsden, Mount Vernon and Nottingham) were kept separate during testing to provide a centre-based testing framework, mirroring real-world evaluation practice

Clinical and histological data were collected from electronic patient records. Patient overall survival was documented up to 3 years post-diagnosis, in line with prior study [14]. It is defined as the time from the baseline diagnostic CT to 3-year follow-up or patient death of any cause, whichever occurred earlier. We excluded cases with tumour histology other than NSCLC, missing clinical data, or suitable imaging data (small tumour volume (< 5 ml) not suitable for [^18^F]FDG PET assessment, or low FDG avidity (SUV_mean_ of such cases: 0.7–2.3) preventing semi-automated segmentation on [^18^F]FDG PET), given the known negative impact of inaccurate image segmentation on radiomic feature reproducibility [21]. CONSORT diagram of the study cohorts and patient characteristics are presented in Fig. 2 and Table 1, respectively.

**Table 1:** Characteristics of patients included in the study and *p*-values showing statistical differences between the discovery and testing cohorts. Notes: Percentage figures are given in brackets, unless otherwise specified. ECOG, Eastern Cooperative Oncology Group; simplified disease stage based on International Association for the Study of Lung Cancer (IASLC) 7th edition; NA: data not available; *p*-values were calculated using two-sided Wilcoxon signed rank test: *denotes statistically significant difference

	Discovery *N* = 285 No. (%)	KCL *N* = 53 No. (%)	*p*-value	Marsden *N* = 63 No. (%)	*p*-value	Mount Vernon *N* = 61 No. (%)	*p*-value	Nottingham *N* = 38 No. (%)	*p*-value
Age (years)									
Median SD Range	67.7 10.1 32–87	65.8 9.2 46–88	0.12	68.1 14.3 42–88	0.23	71.2 9.5 48–91	0.029*	69.7 10.4 53–101	0.50
Sex									
Female Male	114 (40.0) 171 (60.0)	22 (41.5) 31 (58.4)	0.84	20 (31.7) 43 (68.3)	0.22	29 (47.5) 32 (52.5)	0.28	17 (44.7) 21 (55.3)	0.58
ECOG Performance Status									
0 1 2 3 4 Unknown	140 (49.1) 88 (30.8) 39 (13.7) 11 (3.9) 2 (0.7) 5 (1.8)	NA		NA		NA		NA	
T Stage									
1 2 3 4 Unknown	61 (21.4) 118 (41.4) 53 (18.5) 52 (18.2) 1 (0.4)	6 (11.3) 14 (26.4) 15 (28.3) 17 (32.0) 1 (1.9)	0.0013*	11 (17.5) 17 (27.0) 17 (27.0) 18 (28.6)	0.02*	12 (20.7) 25 (43.1) 11 (19.0) 10 (17.2) 3 (4.9)	0.97	3 (7.9) 14 (36.8) 19 (50.0) 2 (5.3)	0.11
N Stage									
0 1 2 3 Unknown	133 (46.7) 46 (16.1) 71 (24.9) 34 (11.9) 1 (0.4)	10 (18.9) 5 (9.4) 33 (62.2) 4 (7.5) 1 (1.9)	0.0018*	26 (41.3) 12 (19.0) 24 (38.1) 1 (1.6)	0.83	22 (38.6) 10 (17.5) 25 (43.9) 1 (1.6) 3 (4.9)	0.58	17 (44.7) 5 (13.2) 11 (28.9) 5 (13.2)	0.63
Metastases									
0 1 Unknown	222 (77.9) 62 (21.7) 1 (0.4)	41 (77.4) 11 (22.6) 1 (1.9)	0.87	62 (98.4) 1 (1.6)	0.00020*	60 (98.4) 0 (0.0) 1 (1.6)	8e-05*	37 (97.4) 1 (2.6)	0.0058*
Histological type									
Squamous Cell Adenocarcinoma Non-specific/Mixed	95 (33.3) 179 (62.8) 11 (3.9)	21 (39.6) 24 (45.3) 8 (15.1)	0.85	25 (39.7) 25 (39.7) 13 (20.6)	0.46	32 (52.4) 20 (32.8) 9 (14.8)	0.12	24 (63.2) 10 (26.3) 4 (10.5)	0.0053*

### Image acquisition

All patients had pre-therapy [^18^F]FDG PET with attenuation correction CT. Patients were examined on different PET/CT scanners as per institutional availability: Discovery cohort (ICHNT)—Siemens Biograph 64 (Siemens Healthcare, Erlangen, Germany); KCL—GE Discovery ST (GE Healthcare, Waukesha, Wisconsin, USA), GE Discovery STE; Marsden—Phillips Allegro Body (Phillips Medical Systems, Amsterdam, Netherlands), Phillips Dual GS, Phillips Gemini TF TOF 16, Siemens Biograph 128; Mount Vernon—Siemens Biograph 64, GE Discovery ST, GE Discovery STE; Nottingham – Siemens Biograph 16.

For PET, slice thickness ranged between 2 and 5.15 mm; matrix size ranged between 128 × 128 and 512 × 512. After injection of 350–500 MBq [^18^F]FDG, emission data were acquired (five or six bed positions, 2–4 min per bed position) after a 60–90 min uptake period. In all cases, PET/CT scans were performed from upper thighs to the base of the skull following ≥ 4–6-h fasting and had a measured blood glucose level < 11.0 mmol/l at the time of injection. A non-contrast CT scan (80–140 mA, 100–140 kVp) was conducted for both attenuation correction of PET data and co-registration with PET images. The PET data were reconstructed using Ordered Subset Expectation Maximization (OSEM) iterative reconstruction and were attenuation-corrected using the CT data.

To account for inter-scanner variability, imaging data were harmonised using voxel resampling and intensity normalisation during pre-processing and ComBat post-radiomics feature extraction [22], in keeping with International Biomarker Standardisation Initiative (IBSI) recommendations [23, 24].

### Multi-label segmentation

**Fig. 3:**
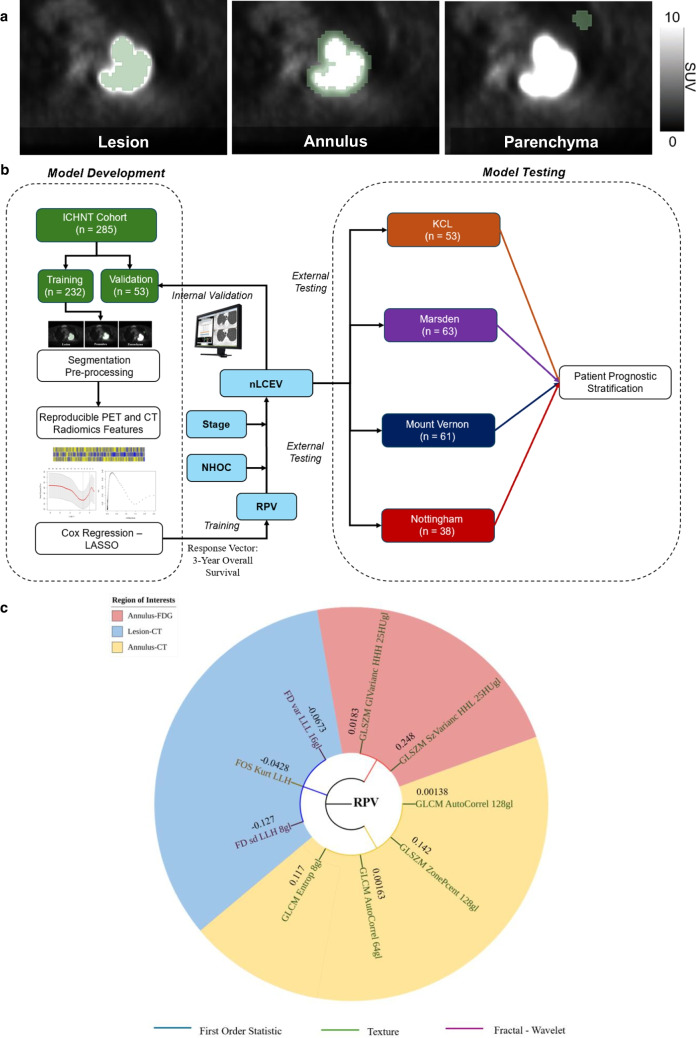
**a**. Multi-label segmentation for radiomics feature extraction showing the three volumes of interests (VOI). **b**. RPV and nLCEV development and testing pipeline. **c**. Constituent radiomic features of RPV: texture features deriving from the annulus VOI on CT followed by first order-wavelet and fractal features from the lesion VOI on CT, and texture-wavelet features from the annulus VOI on [^18^F]FDG PET

Two expert readers, blinded to clinical and histological data, with 13 and 8 years of professional experience, double reviewed all scans, and performed semi-automated segmentation of the primary tumour using metabolic tumour volume 40% SUV_max_ (MTV40) threshold, in keeping with literature recommendation [25]. Additional volumes of interests (VOI) were acquired from the peri-tumoural penumbra as annular shells of 1 cm in thickness, and from the background lung parenchyma as spheres of 2 cm in diameter (Fig. 3a). The peri-tumoural annular shells were generated automatically using an isocontour expansion from the primary lesional VOI. Specifically, after the tumour VOI was defined, consecutive annuli of 1 cm thickness were created outward from the lesion boundary by applying automated three-dimensional iso-contours using an in-house Python script, thereby avoiding manual drawing and inter-observer variability for better reproducibility. The lung parenchymal VOI were manually placed by the radiologist reader in the normal-appearing parenchyma of the ipsilateral lung, cross-referencing the attenuation correction CT scan, with their centres located 2 cm beyond the outer edge of the annulus. Matching VOIs were acquired from the attenuation correction CTs and aggregated with PET features. Parts included in the penumbra and lung VOIs which are anatomically outside of the lungs were excluded by user editing. We have adopted this multi-regional approach for its superior model feature explainability, provided by *post-hoc* feature localisation in the context of intra-tumoural heterogeneity and peri-tumour microenvironment, which is contributing to its growing popularity in NSCLC radiomics literature [14, 26].

All primary tumour delineations were made on 3DSlicer (Slicer Community, Boston, USA) [27], with supplementary VOIs computed using our in-house code implemented in Python 3.7 (Python Software Foundation, Wilmington, USA) and doubly verified by the same two expert readers. To ascertain inter-observer reproducibility, radiomic features was assessed by calculating the intraclass correlation coefficient (ICC), based on a two-way random model and only those with an ICC score ≥ 0.8 were retained, in keeping with literature [21, 23].

### Image processing and radiomic features extraction

After segmentation, the imaging data were pre-processed to ensure uniform voxel size of 3 × 3 × 5 mm and analysed for a total of 3,996 radiomic features from each patient case (666 features per VOI per imaging modality), using an in-house software (TexLab 2.0), implemented in Matlab 2020b (MathWorks Inc., Natick, MA), and previously validated in various studies [11, 14, 28, 29]. Anisotropic voxel resampling was employed to preserve the native spatial resolution of the PET/CT images as much as possible, to minimise resampling-related artefacts and maintain anatomical fidelity, particularly in the z-axis where the voxel size was larger. While the IBSI recommends isotropic resampling for feature standardisation [23], we prioritised retention of native imaging characteristics to ensure robustness across clinically acquired, anisotropically reconstructed datasets.

The choice of using our in-house radiomics tool, TexLab 2.0, is to allow for greater flexibility in implementing region-specific discretisation strategies, tailored filtering operations, and spatial feature localisation, which are not natively supported in standard open-source tools such as PyRadiomics. The in-house pipeline also enabled closer integration with our pre-existing imaging workflow and facilitated enhanced traceability of feature calculations, ensuring consistency across diverse imaging modalities and institutional datasets. The computed features included ones pertaining to FDG avidity, radiodensity, shape, texture, and those from wavelet or Laplacian of Gaussian (LoG) transformed images which are IBSI compliant [23, 24]. We have additionally extracted an additional texture descriptor not yet covered by IBSI, fractal dimension (FD), to capture complex spatial patterns not well characterised by traditional metrics, as demonstrated in several NSCLC radiomics applications [30–32]. This was computed using a box-counting algorithm, which involved overlaying grids of varying box sizes over the VOI and computing the number of boxes required $N(\varepsilon )$ to cover the object as a function of box size $\varepsilon$. FD was then estimated as the negative slope of the linear regression line fitted to the log–log plot of $\mathrm{log}(N\left(\varepsilon \right))$ versus $\mathrm{log}(1/\varepsilon )$.

Following their extraction, the computed radiomic features were standardised to a mean of zero and standard deviation (SD) of one. There were 2,178 features found to have an ICC score ≥ 0.8, thus deemed reproducible and passed onto subsequent model development steps including full dimensionality reduction and regression.

To address collinearity among extracted radiomic features, we computed pairwise Pearson correlation coefficients across all features within the training set. Features exhibiting high linear correlation (|r|> 0.9) were considered redundant; in such cases, one feature from each highly correlated pair was removed based on domain relevance and variance. We then performed univariable Cox regression on each retained radiomic feature, eliminating features with a false detection rate (FDR) of greater than 1%. These filtering steps were performed prior to any model training, to reduce multicollinearity, minimise overfitting, and enhance model interpretability [33]. The resulting subset of 87 filtered features was then standardised (z-score normalised) and used for subsequent predictive modelling.

### Model development and validation

The biomarker development pipeline is presented in Fig. 3b., where we used elastic net regularisation and regression to develop a radiomics predictive vector (RPV) with patient overall survival as the response vector. Using multivariable Cox modelling, this was combined with NHOC and clinical features which were deemed statistically significant on Cox analyses, to develop a composite non-invasive lung cancer evolution vector (nLCEV) for patient prognostication. Biomarker performance was tested by stratifying the patients into a high- and low-risk group using k-means clustering based on nLCEV. Performance was assessed in both the internal and external validation cohorts.

### Normalised hotspot to centroid distance (NHOC)

The Hotspot to Centroid (HOC) distance is calculated as [18]:

$$HOC=\sqrt{{\left({x}_{sm}-{x}_{c}\right)}^{2}+{\left({y}_{sm}-{y}_{c}\right)}^{2}+{\left({z}_{sm}-{z}_{c}\right)}^{2}}$$

where ${x}_{sm}$, ${y}_{sm}$, ${z}_{sm}$ are the spatial coordinates of the voxel with $SU{V}_{max}$; and ${x}_{c}$, ${y}_{c}$, ${z}_{c}$ are those of the geometric centroid of the tumour.

To account for the difference in tumour size, HOC is normalised to MSR, giving normalised HOC (NHOC):

$$NHOC=\frac{HOC}{MSR}$$

where $MSR={\left(\frac{3}{4\pi }MV\right)}^\frac{1}{3}$ for a spherical tumour shape approximation such that metabolic volume (MV): $MV=N\times {V}_{v}$, where $N$ and ${V}_{v}$ are the number of FDG avid voxels and voxel size, respectively.

### Biomarker benchmarking

SUV_max_, MTV, total lesion glycolysis (TLG). and disease stage are known prognostic predictors for NSCLC [34–36]. To establish the novelty of our proposed biomarkers, we investigated how they performed compared to these more conventional prognostic metrics. This includes SUV_max_ and a composite disease stage-metabolic model (SMM), which was imputed based on tumour stage, SUV_max_, MTV, and TLG, using multivariable Cox regression based on the training set.

### Statistical analysis

All statistical analyses were performed using R version 4.3.0 (R Project for Statistical Computing, http://www.r-project.org/). Univariable and multivariable Cox regression models were used to evaluate RPV, NHOC and various clinical features (age, performance status and disease stage) in terms of their relevance to patient survival. Kaplan–Meier plots were used to evaluate the models’ utility for patient prognostication, and log-rank test was used to assess the survival curve differences. Receiver operating characteristics (ROC) analysis was used to assess the predictive performance of RPV and nLCEV and quantified as area under the curve (AUC). A statistical significance threshold of 5% was adopted throughout.

## Results

Comparing to discovery, the testing cohorts were statistically different in at least one element of disease stage; Mount Vernon and Nottingham were additionally statistically different in patient age and NSCLC histological subtype, respectively.

nLCEV is based on NHOC, clinical features and RPV, which is a 9-feature radiomics vector (Fig. 3c), consists of features derived from the three VOIs, including texture features deriving from the annulus VOI on CT, first order-wavelet and fractal features from the lesion VOI on CT, and texture-wavelet features from the annulus VOI on [^18^F]FDG PET.

**Fig. 4:**
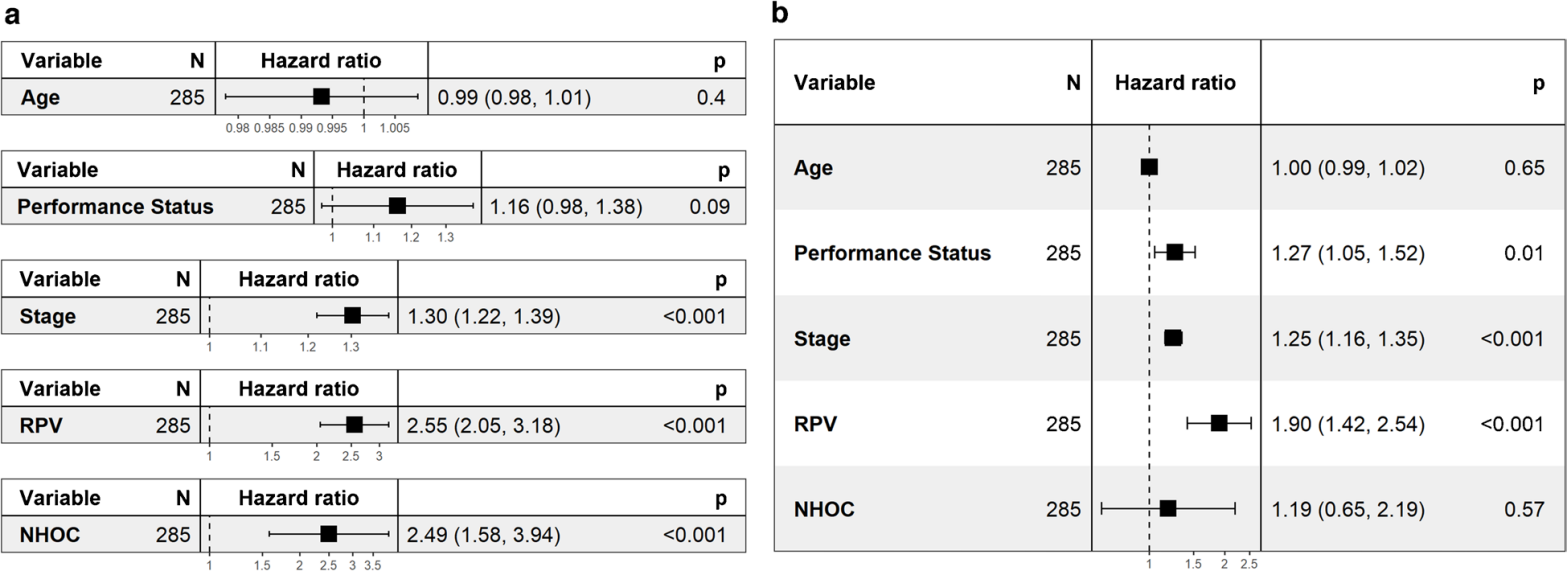
**a**. Univariable and **b**. multivariable Cox regression of key clinical features, RPV and NHOC, showing the statistical significance of tumour stage, RPV and NHOC in univariable Cox and tumour stage and RPV in multivariable Cox models

Univariable and multivariable Cox regression analyses identified clinical features with statistical significance (Fig. 4): tumour staging and RPV were deemed significant (p < 0.05) in both univariable and multivariable regressions and NHOC in univariable regression. Disease staging was therefore used in the development of nLCEV.

**Fig. 5:**
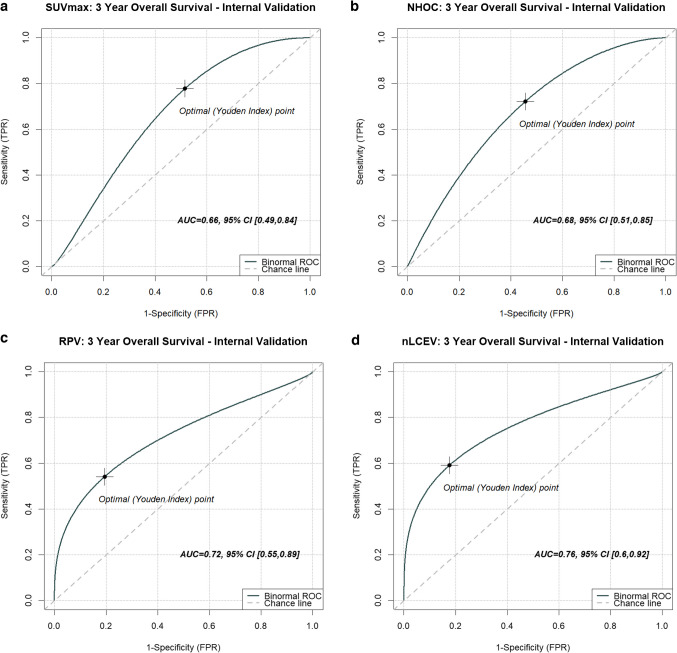
Receiver operating characteristics (ROC) analysis for evaluating the predictive performance for patients’ 3-year overall survival in the internal validation cohort by **a**. SUV_max_, **b**. NHOC, **c**. RPV and **d**. nLCEV; with nLCEV achieving the highest area under the curve (AUC) at 0.76 [0.60, 0.92]

**Fig. 6:**
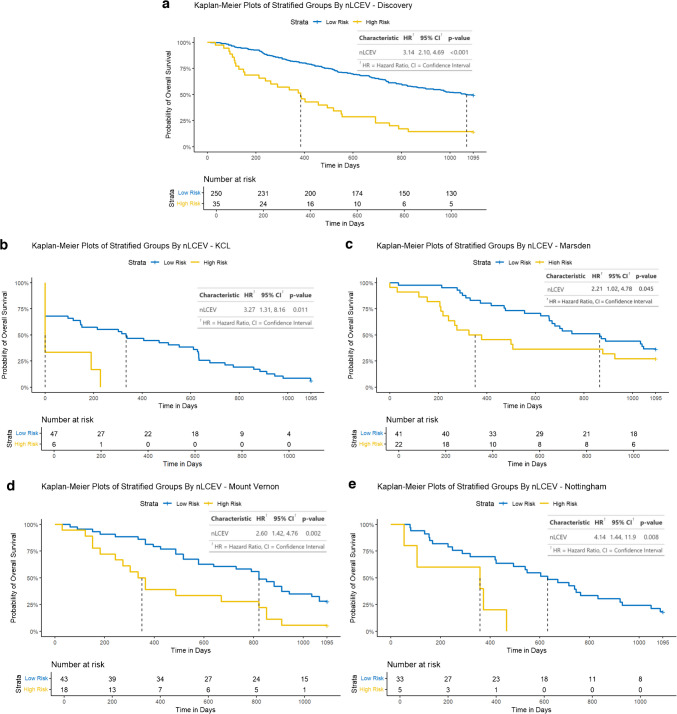
Performance of nLCEV for patient prognostication in the discovery and external validation cohorts: **a**. discovery, **b**. KCL, **c**. Marsden, **d**. Mount Vernon, and **e**. Nottingham. nLCEV achieved patient stratification into high and low risk groups with statistical significance (*p* < 0.05) in all cohorts

**Fig. 7:**
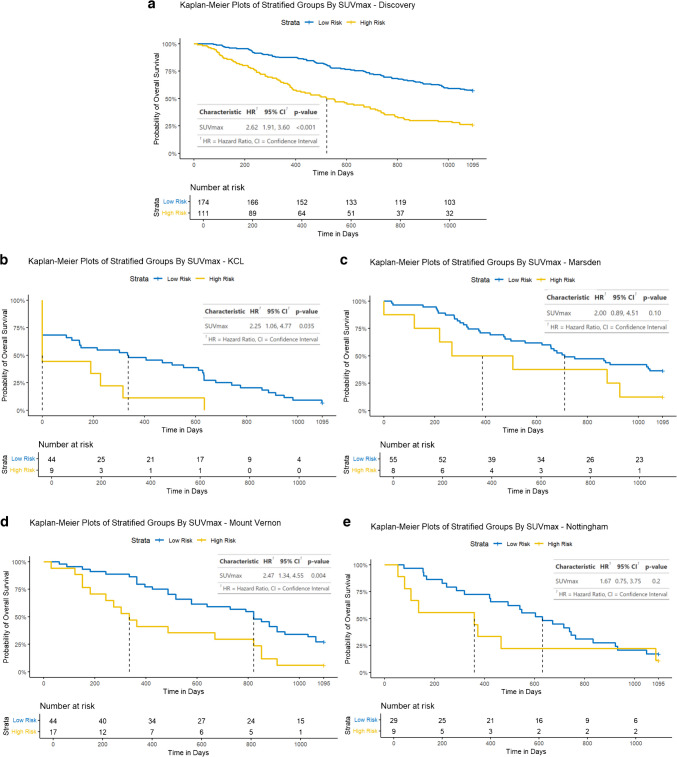
Performance of SUV_max_ for patient prognostication in the discovery and external validation cohorts: **a**. discovery, **b**. KCL, **c**. Marsden, **d**. Mount Vernon, and **e**. Nottingham. In addition to having HRs lower than that of nLCEV, SUV_max_ has not achieved statistically significant prognostic stratification (p < 0.05) in the Marsden and Nottingham cohorts

NHOC and RPV both demonstrated statistically significant Cox hazard ratios (HR): HR [95% confidence interval] 2.52 [1.60, 3.98] and 2.68 [2.13, 3.38], respectively. Neither NHOC nor RPV were strongly correlated with lesional VOI volume to suggest intermetric surrogacy, with a Pearson’s correlation coefficient (PCC) of 0.404 and 0.583, respectively. NHOC produced an area under the receiver operating characteristic curve (AUC) [95% confidence interval] of 0.68 [0.51, 0.85] for predicting patients’ overall survival at 3 years, RPV 0.72 [0.55, 0.89] and nLCEV the highest AUC at 0.76 [0.60, 0.92], compared to that of SUV_max_ 0.66 [0.49, 0.84] (Fig. 5). Statistically significant stratification of the patients into high and low risk groups was achieved using nLCEV in the discovery and all external testing cohorts (*p*-value < 0.05, Fig. 6), with reasonable Cox hazard ratios (HR): discovery 3.14 [2.10, 4.69], KCL 3.27 [1.31, 8.16], Marsden 2.21 [1.02, 4.78], Mount Vernon 2.60 [1.42, 4.76], and Nottingham 4.14 [1.44, 11.90], which are consistently higher than the corresponding HRs achieved using SUV_max_ (Fig. 7). Additionally, SUV_max_ has not achieved statistically significant prognostic stratification in the Marsden and Nottingham cohorts (Fig. 7c and e).

**Fig. 8:**
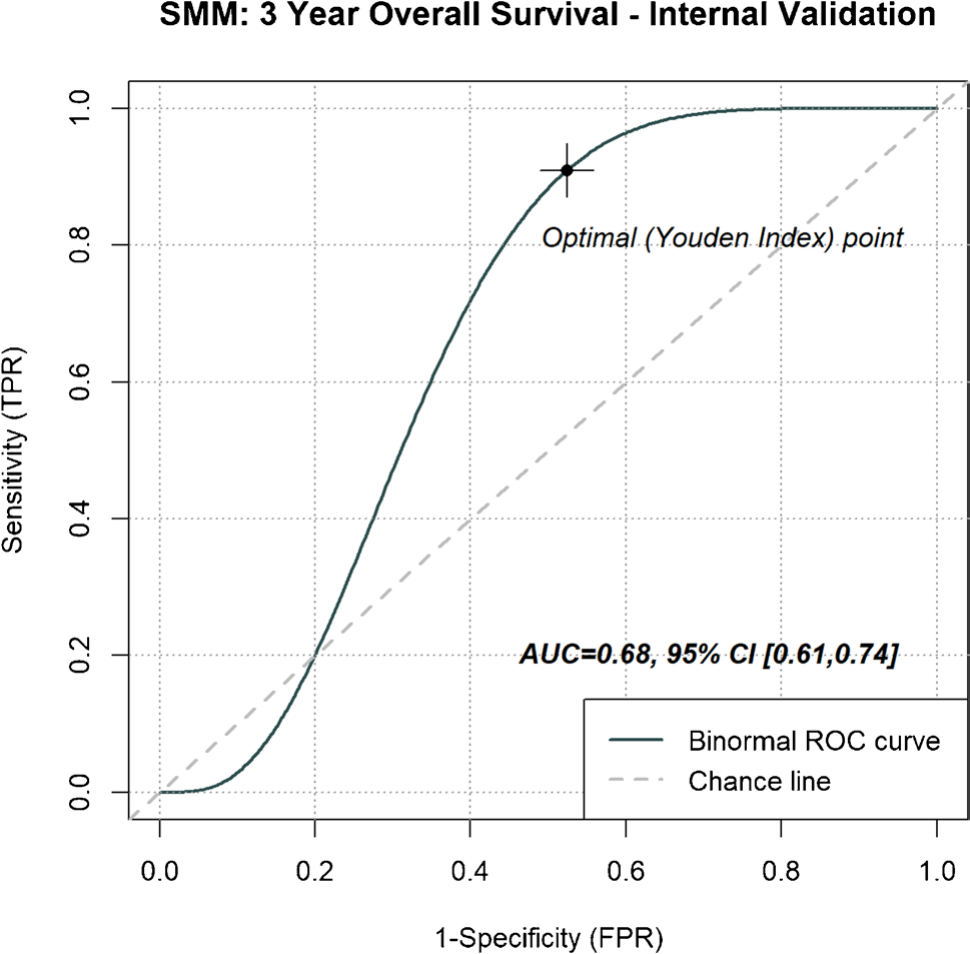
Receiver operating characteristics (ROC) analysis for evaluating the predictive performance for patients’ 3-year overall survival in the internal validation cohort by SMM. With an area under the curve (AUC) of 0.68 (95% CI [0.61, 0.74]); SMM was inferior to nLCEV’s AUC of 0.76 (95%CI [0.60, 0.92])

**Fig. 9:**
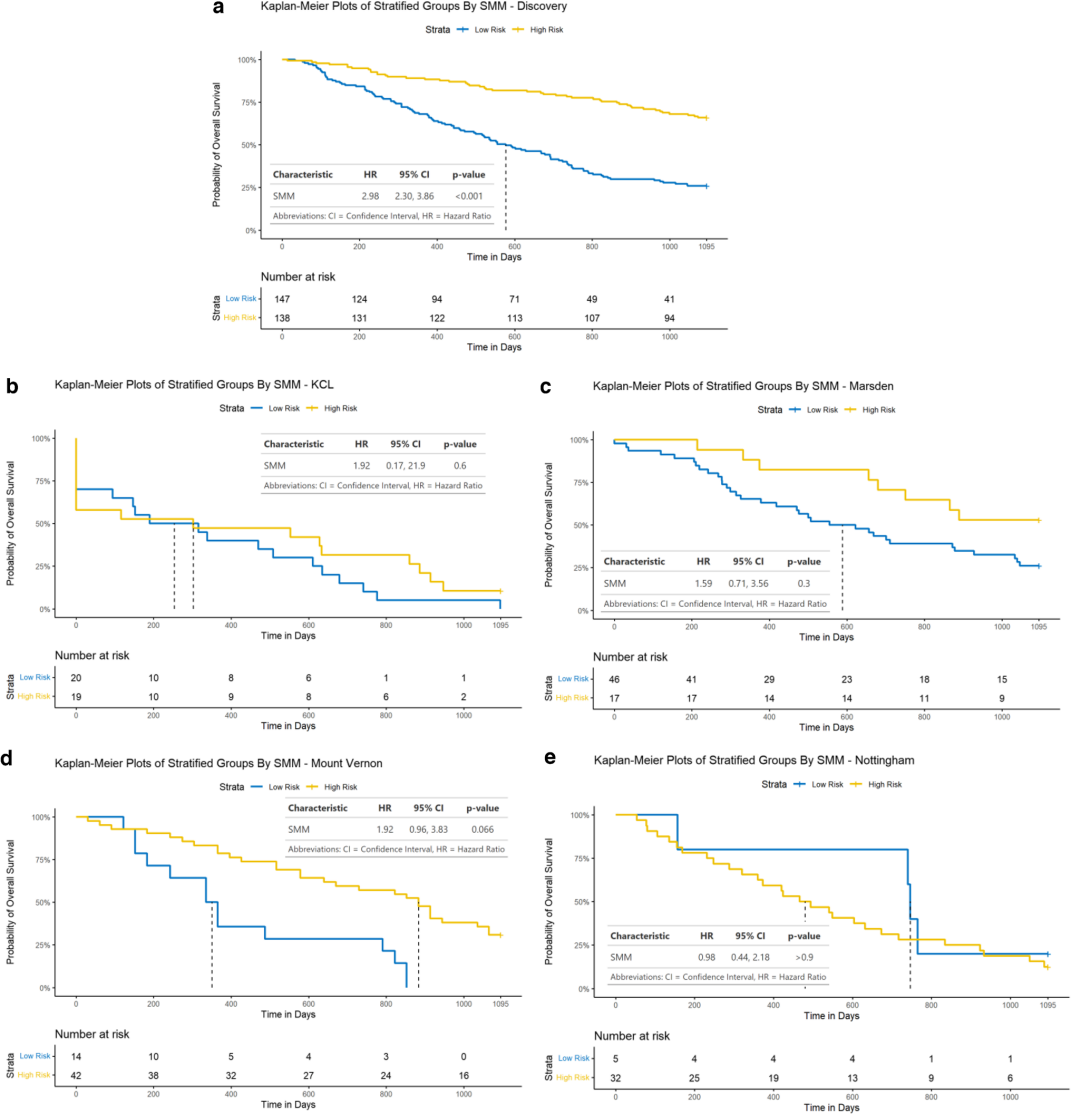
Performance of disease stage-metabolic model (SMM) for patient prognostication in the discovery and external validation cohorts: **a**. discovery, **b**. KCL, **c**. Marsden, **d**. Mount Vernon, and **e**. Nottingham. Whilst achieving effective prognostic stratification in discovery, it has failed to achieve similar performance in the external validation cohorts

Whilst SMM has achieved an AUC of 0.68 (95% CI [0.61, 0.74]) (Fig. 8), it remains inferior to nLCEV’s 0.76 (95%CI [0.60, 0.92]). More notably, similar to what we had observed with SUV_max_, SMM demonstrated inferior robustness compared to nLCEV, as it failed to achieve effective prognostic stratification in the external validation cohorts (KCL: *p* = 0.6, Marsden: *p* = 0.3, Mount Vernon: *p* = 0.066, Nottingham: *p* > 0.9; Fig. 9).

## Discussion

Most known prognostic radiomic features are not derived from comprehensible cancer phenotypes and thus carry limited causal justification to support their biological validity, which has been highlighted as a key burden to their clinical adoption [37–39]. In this study, we have demonstrated in NSCLC the prognostic utility of NHOC, a cancer biology–inspired metric of spatial differences in tumour architecture. Building on this concept, we developed a composite non-invasive lung cancer prognostic vector, nLCEV, by aggregating the prognostic power of NHOC with that of [^18^F]FDG PET and CT radiomics, and tumour staging. Specifically, fractal and wavelet [^18^F]FDG PET features from the perilesional annulus, CT texture features from the lesion and perilesional annulus were retained in the radiomics prognostic signature. NHOC and RPV were both significant in Cox regression models, alongside tumour stage; these were integrated in nLCEV, which outperforms both RPV and NHOC and achieved effective patient prognostication in all four independent external testing sets. The external testing sets have various statistically significant different patient and disease characteristics to those of the discovery cohort; the successful validation of the biomarkers in these cohorts therefore supports their overall robustness. The observed distributional differences between discovery and testing cohorts provide a more rigorous assessment of model generalisability across diverse clinical settings. Evaluating performance on cohorts that differ from the training set in clinically relevant variables helps to identify potential overfitting and ensures the robustness of predictive features beyond the original dataset. This approach also mirrors real-world deployment scenarios, where patient populations, imaging protocols, and disease characteristics often vary across institutions. Through this work, we have additionally developed a software for computer-assisted multi-label VOI segmentation and feature extraction on PET-CT, to aid in the clinical deployment of the innovations this paper presents.

The differences observed between the survival characteristics of the Marsden and Nottingham cohorts (Figs. 6, 7 and 9) might be attributable to their variations in tumour stage and histological composition. The Marsden cohort included more patients with both early (T1: 17.5% vs. 7.9%) and advanced (T4: 28.6% vs. 5.3%) disease compared to Nottingham. Additionally, there were differences in histological subtype distribution, with fewer squamous cell carcinomas (39.7% vs. 63.2%) and more adenocarcinomas (39.7% vs. 26.3%) in the Marsden cohort. These differences in case mix likely contributed to the varied results observed in these cohorts.

Prior studies have proposed MTV-derived tumour volume and SUV_max_ as prognostic predictors in NSCLC [40–43]. In our work, we found no strong correlation between our evaluated prognostic metrics (NHOC, RPV) and tumour volume by MTV40 to suggest inter-metric surrogacy. Additionally, these imaging metrics demonstrated better prognostic prediction of patient survival than that by SUV_max_ (Fig. 5), and higher HR in the prognostic stratification of patient risk groups (Figs. 6 and 7). The best performing biomarker, nLCEV, incorporates disease stage, itself a well-established prognostic factor [36]. The demonstrated superiority of nLCEV over SMM (Figs. 8 and 9), a multivariable signature derived from stage, SUV_max_, MTV, and TLG, shows that nLCEV provides added prognostic value beyond what can be achieved by conventional clinical and imaging metrics alone.

Previously, [^18^F]FDG PET metrics found to be the most prognostic significance included ${\mathrm{SUV}}_{max}$ and TLG, which were associated with shorter progression-free survival (PFS) and OS [44]; texture-entropy, which was associated with poorer response to treatment [45], and MTV, which correlated with worse patient prognosis and was thought to suggest higher tumour burden [46]. In our study, we found two enriched [^18^F]FDG PET radiomic features, both are wavelet-transformed texture features extracted from the peri-lesional annulus VOI, one of which, *GLSZM SzVarianc HHL*, carries the highest weight of all constituent features in RPV. These observations are concordant with the underlying hypothesis behind NHOC, such that the clonal drift of cancer cells towards the tumour periphery is associated with a worse patient prognosis [47]. Additionally, the greatest number of the retained features (*n* = 4) belonged to the texture feature class from the peri-lesional annulus VOI on CT, which would support a hypothesised peripheral distribution of the most prognostic cancer cells. This is further reinforced by the observation that all enriched features derived from the peri-lesional VOI on [^18^F]FDG PET and CT carried positive weights whereas the three features derived from the lesion VOI on CT carried negative weights, which proposes a positive dependency of patient mortality on the former and a negative one on the latter.

Literature on using [^18^F]FDG PET/CT radiomics for NSCLC prognostication has expanded rapidly in recent years [38, 48]. For surgical cases, pre-operative PET/CT radiomics have predicted disease-free survival to an AUC of 0.68 [0.58, 0.74] [49]. Pre-therapy PET radiomics predicted the survival of metastatic NSCLC to an AUC of 0.70 [50]. Despite the relative abundance of radiomics literature, few studies have presented models validated on independent test data, and even rarer are those validated on data drawn from multiple sources [11], a key strength of nLCEV. Comparing to an earlier work drawn from similar data [11], our proposed composite biomarker, nLCEV, was developed without training–testing data mixing, demonstrated site-specific robustness, with effective prognostication in multiple external validation datasets individually, as well as offering better biological explainability.

Imaging-based metrics such as radiomics offer a non-invasive way of assessing neoplasms at the time of diagnosis. This can aid in clinical decision making, particularly in cases where tissue sampling is challenging or yields equivocal findings. The information presented by these measurements can additionally give insight into tumour composition and metabolism, thereby advancing an understanding of cancer biology that is not otherwise achievable *in vivo*. Compared to conventional radiomics practice dominated by data mining of a high-order data space constructed from multi-class multi-label radiomic features, which can be burdened by feature redundancy, the curse of dimensionality and risk of overfitting [4], our method of incorporating NHOC, a novel mathematical descriptor of intratumoural spatial organisation motivated by cancer biology, introduces an element of causal reasoning [51], which helps to avoid spurious conclusions arising out of confounding or less biologically meaningful statistical associations. Before undertaking their testing in prospective randomised interventional trials, this approach can also enable the prediction of outcomes to hypothetical interventions or counterfactuals, such as by predicting the efficacy of imaging/machine learning-supported treatment decisions.

In contrast to conventional, or *hand-crafted*, radiomics, deep learning-derived features are gaining prominence in recent literature due to their ability to automatically learn complex, high-dimensional representations from imaging data without the need for manual feature engineering [52–54]. However, they lack the interpretability of their hand-crafted counterparts [52]. To mitigate this limitation, *post-hoc* methods such as saliency maps, SHapley Additive exPlanations (SHAP), Local Interpretable Model-agnostic Explanations (LIME), and Gradient-weighted Class Activation Mapping (Grad-CAM) can be employed [55], some of which can also be applied to less interpretable models involving hand-crafted radiomics features [56, 57]. While these techniques can highlight associations between input features and model predictions, they do not necessarily provide mechanistic insight or causal linkage to disease processes [58]. Departing from purely data-driven strategies, our approach incorporates a biologically grounded imaging metric, NHOC, shifting the emphasis from correlation-based interpretation to hypothesis-driven feature design, a distinction that is critical for enhancing model transparency and trustworthiness [59]. Our method notably delivers explainability by embedding biological justification at the feature construction stage, rather than relying solely on retrospective model interrogation.

Although NHOC was originally postulated to reflect clonal competition between neoplastic subpopulations, it does not allow their direct visualisation, which would likely require additional imaging tracers. While motivated by concepts of cancer biology, NHOC should not yet be regarded as a validated biology-derived metric, but rather as an imaging-based surrogate of spatial heterogeneity holding prognostic relevance. Additional limitations of this study include its retrospective nature and the relatively small size of the external testing sets, warranting wider scale external validation. The study is also limited by the exclusion of cases with low [^18^F]FDG avidity barring reliable segmentation on PET. This latter group represented adenocarcinoma cases with generally longer observed survival than the rest [60]. On this note, we acknowledge the marked clinical and histological heterogeneity of adenocarcinoma-spectrum disease, not all of which can be reliably and prognostically assessed on [^18^F]FDG PET, a notable limitation of the modality in this clinical context [61]. Radiomic features can be affected by the type of CT scanner and scanning protocol used [62]. However, we have previously shown an [^18^F]FDG PET/CT derived radiomics feature vector to be invariant to PET/CT scanner type and slice thickness [11]. We have further ascertained feature reproducibility in this study by including only features meeting an ICC score threshold. To verify its generalisability and robustness, the biomarker was validated on external testing data acquired in four independent institutions with different scanners and scanning protocols, and varied patient and disease characteristics.

Future works include testing the biomarkers prospectively and evaluating its utility in clinical practice, integration with other validated imaging prognostic metrics in cancer such as sarcopenia [63], delta radiomics [64], and cancer biology correlates through spatial and transcriptomics mapping [65]. Additionally, intra-tumoural biological heterogeneity is becoming increasingly recognised for its key role in determining therapeutic response and resistance in NSCLC [66, 67]. We have conducted in an internally validated pilot study demonstrating the potential utility of integrating histological tumour features to enhance the performance of nLCEV (see Supplementary materials). Future work could therefore also include collecting and integrating biological measures of intra-tumoural heterogeneity, from histological and molecular domains, to deliver better prognostic biomarkers with enhanced biological explainability.

A limitation of this study is that the imaging metrics examined, including NHOC, SUVmax, MTV, and TLG, were derived solely from the primary tumour. This approach does not account for metastatic disease burden or inter-lesional heterogeneity, both of which are becoming increasingly recognised as important prognostic factors in NSCLC [68]. Recent advances in AI-based PET/CT analysis allow for automated, whole-body quantification of MTV and TLG across all metastatic sites [69, 70]. Incorporating such whole-body metrics may provide a more complete representation of tumour biology and patient prognosis. Our proposed framework could, in principle, be extended to include all metastatic lesions, and future work could focus on evaluating its utility in this broader context.

In conclusion, NHOC enhances NSCLC patient survival prediction, and when integrated with PET-CT radiomics and disease stage, offers a robust, non-invasive approach to disease prognostication, with consistent performance demonstrated across multiple independent external cohorts.

## Supplementary Information

Below is the link to the electronic supplementary material.

Supplementary file1 (DOCX 1.09 MB)

## Data Availability

The study data (clinical and imaging) are retrospective in nature and protected through institutional compliance; they can be shared as per specific institutional review board (IRB) requirements. Upon reasonable request, a data sharing agreement can be initiated between the interested parties and the clinical institution following institution-specific guidelines.

## Code availability

All R scripts and guidance for use can be found on GitHub: https://github.com/scat2801/nhoc-rpv/.

## References

[1] Cancer Research UK. Types of lung cancer [Internet]. 2019. [cited 2021 Aug 23]. https://www.cancerresearchuk.org/about-cancer/lung-cancer/stages-types-grades/types. Accessed 23 Aug 2021.

[2] Polanco D, Pinilla L, Gracia-Lavedan E, Mas A, Bertran S, Fierro G, et al. Prognostic value of symptoms at lung cancer diagnosis: a three-year observational study. J Thorac Dis [Internet]. AME Publications; 2021 [cited 2024 Sep 24];13:1485. 10.21037/JTD-20-3075. 10.21037/jtd-20-3075 PMC8024804 33841941

[3] Lambin P, Rios-Velazquez E, Leijenaar R, Carvalho S, Van Stiphout RGPM, Granton P, et al. Radiomics: extracting more information from medical images using advanced feature analysis. Eur J Cancer. 2012;48:441–6. 10.1016/J.EJCA.2011.11.036. 22257792 10.1016/j.ejca.2011.11.036 PMC4533986

[4] Chen M, Copley SJ, Viola P, Lu H, Aboagye EO, Academic Press. Radiomics and artificial intelligence for precision medicine in lung cancer treatment. Semin Cancer Biol. 2023;93:97–113. 10.1016/J.SEMCANCER.2023.05.004. 37211292 10.1016/j.semcancer.2023.05.004

[5] Zhang Y, Oikonomou A, Wong A, Haider MA, Khalvati F. Radiomics-based prognosis analysis for non-small cell lung cancer. Sci Rep. 2017. 10.1038/SREP46349. 28418006 10.1038/srep46349 PMC5394465

[6] Cho H ho, Lee HY, Kim E, Lee G, Kim J, Kwon J, et al. Radiomics-guided deep neural networks stratify lung adenocarcinoma prognosis from CT scans. Commun Biol [Internet]. Nature Publishing Group; 2021 [cited 2023 Apr 12];4:1–12. 10.1038/s42003-021-02814-7. 10.1038/s42003-021-02814-7 PMC8590002 34773070

[7] Kang W, Qiu X, Luo Y, Luo J, Liu Y, Xi J, et al. Application of radiomics-based multiomics combinations in the tumor microenvironment and cancer prognosis. J Transl Med [Internet]. BioMed Central Ltd; 2023 [cited 2024 Feb 4];21:1–20. 10.1186/S12967-023-04437-4/FIGURES/3. 10.1186/s12967-023-04437-4 PMC10481579 37674169

[8] Hannequin P, Decroisette C, Kermanach P, Berardi G, Bourbonne V. FDG PET and CT radiomics in diagnosis and prognosis of non-small-cell lung cancer. Transl Lung Cancer Res [Internet]. AME Publishing Company; 2022 [cited 2023 Nov 5];11:2051–63. 10.21037/TLCR-22-158/COIF. 10.21037/tlcr-22-158 PMC9641045 36386457

[9] Wu L, Lou X, Kong N, Xu M, Gao C, Springer Science and Business Media Deutschland GmbH. Can quantitative peritumoral CT radiomics features predict the prognosis of patients with non-small cell lung cancer? A systematic review. Eur Radiol. 2022. 10.1007/S00330-022-09174-8. 36307554 10.1007/s00330-022-09174-8 PMC9935659

[10] Al Doori L, Evanson D, Revheim M-E, Saboury B, Alavi A, Torigian D. Advancements in [18F]FDG-PET/CT radiomics and machine learning for non-small cell lung cancer. J Nucl Med [Internet]. Society of Nuclear Medicine; 2024 [cited 2025 Jan 13];65:241765–241765. https://jnm.snmjournals.org/content/65/supplement_2/241765. Accessed 13 Jan 2025.

[11] Arshad MA, Thornton A, Lu H, Tam H, Wallitt K, Rodgers N, et al. Discovery of pre-therapy 2-deoxy-2-(18)F-fluoro-D-glucose positron emission tomography-based radiomics classifiers of survival outcome in non-small-cell lung cancer patients. Eur J Nucl Med Mol Imaging. 2019;46:455–66. 10.1007/s00259-018-4139-4. 30173391 10.1007/s00259-018-4139-4 PMC6333728

[12] Boubnovski Martell M, Linton-Reid K, Hindocha S, Chen M, Moreno P, Álvarez‐Benito M, et al. Deep representation learning of tissue metabolome and computed tomography annotates NSCLC classification and prognosis. NPJ Precis Oncol [Internet]. Nature Publishing Group; 2024 [cited 2024 Feb 4];8:1–14. 10.1038/s41698-024-00502-3. 10.1038/s41698-024-00502-3 PMC10838282 38310164

[13] Lu H, Lou H, Wengert G, Paudel R, Patel N, Desai S, et al. Tumor and local lymphoid tissue interaction determines prognosis in high-grade serous ovarian cancer. Cell Rep Med. Cell Press; 2023;4:101092. 10.1016/J.XCRM.2023.101092. 10.1016/j.xcrm.2023.101092 PMC10394173 37348499

[14] Chen M, Lu H, Copley SJ, Han Y, Logan A, Viola P, et al. A novel radiogenomics biomarker for predicting treatment response and pneumotoxicity from programmed cell death protein or Ligand-1 inhibition immunotherapy in NSCLC. J Thor Oncol [Internet]. Elsevier Inc.; 2023 [cited 2023 Oct 6];18:718–30. 10.1016/j.jtho.2023.01.089. 10.1016/j.jtho.2023.01.089 36773776

[15] Lv X, Mao Z, Sun X, Liu B. Intratumoral heterogeneity in lung cancer. Cancers (Basel). 2023;15:2709. 10.3390/CANCERS15102709. 37345046 10.3390/cancers15102709 PMC10216154

[16] Weir-McCall JR, Joyce S, Clegg A, MacKay JW, Baxter G, Dendl LM, et al. Dynamic contrast–enhanced computed tomography for the diagnosis of solitary pulmonary nodules: a systematic review and meta-analysis. Eur Radiol [Internet]. Springer; 2020 [cited 2025 Jan 3];30:3310–23. 10.1007/S00330-020-06661-8/TABLES/5. 10.1007/s00330-020-06661-8 32060716

[17] Vanhove K, Graulus GJ, Mesotten L, Thomeer M, Derveaux E, Noben JP, et al. The metabolic landscape of lung cancer: new insights in a disturbed glucose metabolism. Front Oncol [Internet]. Frontiers Media S.A.; 2019 [cited 2025 Jan 3];9:492161. 10.3389/FONC.2019.01215/BIBTEX. 10.3389/fonc.2019.01215 PMC6873590 31803611

[18] Jiménez-Sánchez J, Bosque JJ, Jiménez Londoño GA, Molina-García D, Martínez Á, Pérez-Beteta J, et al. Evolutionary dynamics at the tumor edge reveal metabolic imaging biomarkers. Proc Natl Acad Sci U S A [Internet]. National Academy of Sciences; 2021 [cited 2022 Nov 27];118:e2018110118. 10.1073/pnas.2018110118. 10.1073/pnas.2018110118 PMC8017959 33536339

[19] Soussan M, Orlhac F, Boubaya M, Zelek L, Ziol M, Eder V, et al. Relationship between tumor heterogeneity measured on FDG-PET/CT and pathological prognostic factors in invasive breast cancer. PLoS One [Internet]. Public Library of Science; 2014;9:e94017–e94017. 10.1371/journal.pone.0094017. 10.1371/journal.pone.0094017 PMC3983104 24722644

[20] Cook GJR, Yip C, Siddique M, Goh V, Chicklore S, Roy A, et al. Are pretreatment 18 F-FDG PET tumor textural features in non-small cell lung cancer associated with response and survival after chemoradiotherapy? J Nucl Med. 2013;54:1–8. 10.2967/jnumed.112.107375. 10.2967/jnumed.112.107375 23204495

[21] Traverso A, Wee L, Dekker A, Gillies R, Elsevier. Repeatability and reproducibility of radiomic features: a systematic review. Int J Radiat Oncol Biol Phys. 2018;102:1143–58. 10.1016/J.IJROBP.2018.05.053. 30170872 10.1016/j.ijrobp.2018.05.053 PMC6690209

[22] Mahon RN, Ghita M, Hugo GD, Weiss E. ComBat harmonization for radiomic features in independent phantom and lung cancer patient computed tomography datasets. Phys Med Biol [Internet]. IOP Publishing; 2020 [cited 2022 Nov 15];65:015010. 10.1088/1361-6560/AB6177. 10.1088/1361-6560/ab6177 31835261

[23] Zwanenburg A, Vallières M, Abdalah MA, Aerts HJWL, Andrearczyk V, Apte A, et al. The image biomarker standardization initiative: Standardized quantitative radiomics for high-throughput image-based phenotyping. Radiology [Internet]. Radiological Society of North America Inc.; 2020 [cited 2023 May 13];295:328–38. 10.1148/RADIOL.2020191145/ASSET/IMAGES/LARGE/RADIOL.2020191145.FIG5.JPEG. 10.1148/radiol.2020191145 PMC7193906 32154773

[24] Whybra P, Zwanenburg A, Andrearczyk V, Schaer R, Apte AP, Ayotte A, et al. The image biomarker standardization initiative: Standardized convolutional filters for reproducible radiomics and enhanced clinical insights. Radiology [Internet]. Radiological Society of North America Inc.; 2024 [cited 2025 Apr 30];310. 10.1148/RADIOL.231319. 10.1148/radiol.231319 PMC10902595 38319168

[25] Konert T, Everitt S, la Fontaine MD, van de Kamer JB, MacManus MP, Vogel WV, et al. Robust, independent and relevant prognostic 18F-fluorodeoxyglucose positron emission tomography radiomics features in non-small cell lung cancer: are there any? PLoS One. 2020;15(2):e0228793. 10.1371/JOURNAL.PONE.0228793. 32097418 10.1371/journal.pone.0228793 PMC7041813

[26] Sun R, Limkin EJ, Vakalopoulou M, Dercle L, Champiat S, Han SR, et al. A radiomics approach to assess tumour-infiltrating CD8 cells and response to anti-PD-1 or anti-PD-L1 immunotherapy: an imaging biomarker, retrospective multicohort study. Lancet Oncol. Lancet Publishing Group; 2018;19:1180–91. 10.1016/S1470-2045(18)30413-3. 10.1016/S1470-2045(18)30413-3 30120041

[27] Fedorov A, Beichel R, Kalpathy-Cramer J, Finet J, Fillion-Robin J-C, Pujol S, et al. 3D slicer as an image computing platform for the quantitative imaging network. Magn Reson Imaging. 2012;30:1323–41. 10.1016/j.mri.2012.05.001. 22770690 10.1016/j.mri.2012.05.001 PMC3466397

[28] Hunter B, Chen M, Ratnakumar P, Alemu E, Logan A, Linton-Reid K, et al. A radiomics-based decision support tool improves lung cancer diagnosis in combination with the Herder score in large lung nodules. EBioMedicine [Internet]. Elsevier B.V.; 2022 [cited 2023 Oct 6];86. 10.1016/j.ebiom.2022.104344. 10.1016/j.ebiom.2022.104344 PMC9664396 36370635

[29] Lu H, Arshad M, Thornton A, Avesani G, Cunnea P, Curry E, et al. A mathematical-descriptor of tumor-mesoscopic-structure from computed-tomography images annotates prognostic- and molecular-phenotypes of epithelial ovarian cancer. Nat Commun [Internet]. Nature Publishing Group; 2019 [cited 2022 Nov 16];10:1–11. 10.1038/s41467-019-08718-9. 10.1038/s41467-019-08718-9 PMC6377605 30770825

[30] Amador-Legon NV, Perez-Diaz M. Use of fractals in determining the malignancy degree of lung nodules. Front Med Technol [Internet]. Frontiers Media SA; 2024 [cited 2025 May 5];6:1362688. 10.3389/FMEDT.2024.1362688/FULL. 10.3389/fmedt.2024.1362688 PMC11002126 38595696

[31] Lennon FE, Cianci GC, Cipriani NA, Hensing TA, Zhang HJ, Chen CT, et al. Lung cancer-a fractal viewpoint. Nat Rev Clin Oncol. Nature Publishing Group; 2015;12:664–75. 10.1038/NRCLINONC.2015.108. 10.1038/nrclinonc.2015.108 PMC4989864 26169924

[32] Patil R, Srinidhi Bhat GM, Leonard Wee DMS, Dekker A. Fractal analysis in histology classification of non-small cell lung cancer. Medical Imaging [Internet]. CRC Press; 2019 [cited 2022 Nov 11];62–73. 10.1201/9780429029417-4.

[33] Chen M, Linton-Reid K, Aboagye EO, Copley SJ. Translating radiomics into clinical practice: a step-by-step guide to study design and evaluation. Clin Radiol. 2025. 10.1016/J.CRAD.2025.107053. 40962646 10.1016/j.crad.2025.107053

[34] Takeda A, Sanuki N, Fujii H, Yokosuka N, Nishimura S, Aoki Y, et al. Maximum standardized uptake value on FDG-PET is a strong predictor of overall and disease-free survival for non–small-cell lung cancer patients after stereotactic body radiotherapy. J Thorac Oncol. 2014;9(1):65–73. 10.1097/JTO.0000000000000031. 24346094 10.1097/JTO.0000000000000031

[35] Wen W, Piao Y, Xu D, Li X. Prognostic value of MTV and TLG of ^18^F-FDG PET in patients with stage I and II non-small-cell lung cancer: a meta-analysis. Contrast Media Mol Imaging. 2021;2021:1. 10.1155/2021/7528971. 10.1155/2021/7528971 PMC8629622 34887713

[36] Rami-Porta R, Nishimura KK, Giroux DJ, Detterbeck F, Cardillo G, Edwards JG, et al. The international association for the study of lung cancer lung cancer staging project: Proposals for revision of the TNM Stage Groups in the Forthcoming (Ninth) Edition of the TNM Classification for Lung Cancer. J Thor Oncol [Internet]. Elsevier Inc.; 2024 [cited 2025 Sep 19];19:1007–27. 10.1016/j.jtho.2024.02.011. 10.1016/j.jtho.2024.02.011 38447919

[37] Brady AP, Allen B, Chong J, Kotter E, Kottler N, Mongan J, et al. Developing, purchasing, implementing and monitoring AI tools in radiology: Practical considerations. A multi-society statement from the ACR, CAR, ESR, RANZCR and RSNA. Radiol Artif Intell [Internet]. Radiological Society of North America Inc.; 2024 [cited 2025 Jun 27];6. 10.1148/RYAI.230513/ASSET/IMAGES/LARGE/RYAI.230513.FIG1.JPEG. 10.1148/ryai.230513 PMC10831521 38251899

[38] Zhang Y, Huang W, Jiao H, Kang L. PET radiomics in lung cancer: advances and translational challenges. EJNMMI Phys 2024 11:1 [Internet]. SpringerOpen; 2024 [cited 2025 Feb 3];11:1–29. 10.1186/S40658-024-00685-5. 10.1186/s40658-024-00685-5 PMC11450131 39361110

[39] Huang EP, O’Connor JPB, McShane LM, Giger ML, Lambin P, Kinahan PE, et al. Criteria for the translation of radiomics into clinically useful tests. Nat Rev Clin Oncol 2022 20:2 [Internet]. Nature Publishing Group; 2022 [cited 2025 Apr 21];20:69–82. 10.1038/s41571-022-00707-0. 10.1038/s41571-022-00707-0 PMC9707172 36443594

[40] Yan H, Wang R, Zhao F, Zhu K, Jiang S, Zhao W, et al. Measurement of tumor volume by PET to evaluate prognosis in patients with advanced non-small cell lung cancer treated by non-surgical therapy [Internet]. SAGE PublicationsSage UK: London, England; 2011 [cited 2025 Feb 9];52:646–50. 10.1258/AR.2011.100462. 10.1258/ar.2011.100462 21508201

[41] Berghmans T, Dusart M, Paesmans M, Hossein-Foucher C, Buvat I, Castaigne C, et al. Primary tumor standardized uptake value (SUVmax) Measured on fluorodeoxyglucose positron emission tomography (FDG-PET) is of Prognostic value for survival in non-small cell lung cancer (NSCLC): A systematic review and meta-analysis (MA) by the European lung cancer working party for the IASLC lung cancer staging project. J Thor Oncol. Elsevier; 2008;3:6–12. 10.1097/JTO.0B013E31815E6D6B. 10.1097/JTO.0b013e31815e6d6b 18166834

[42] Zhao K, Wang C, Shi F, Huang Y, Ma L, Li M, et al. Combined prognostic value of the SUVmax derived from FDG-PET and the lymphocyte-monocyte ratio in patients with stage IIIB-IV non-small cell lung cancer receiving chemotherapy. BMC Cancer [Internet]. BioMed Central Ltd; 2021 [cited 2025 Feb 8]; 21:1–13. 10.1186/S12885-021-07784-X/TABLES/6. 10.1186/s12885-021-07784-x PMC7809816 33446134

[43] Deshpande S, Podder TK, Zhang Y, Zheng Y, Grubb W, Kharouta M, et al. Prognostic value of SUVmax on FDG-PET/CT before and after stereotactic body radiotherapy (SBRT) on recurrence and survival in early-stage non-small cell lung cancer (NSCLC). J Clin Oncol [Internet]. Wolters Kluwer Health; 2021 [cited 2025 Feb 8];39:8539–8539. 10.1200/JCO.2021.39.15_SUPPL.8539.

[44] Liu J, Dong M, Sun X, Li W, Xing L, Yu J, et al. Prognostic value of 18F-FDG PET/CT in surgical non-small cell lung cancer: a meta-analysis. PLoS One. 2016;11:e0146195. 10.1371/JOURNAL.PONE.0146195. 26727114 10.1371/journal.pone.0146195 PMC4699812

[45] Pyka T, Bundschuh RA, Andratschke N, Mayer B, Specht HM, Papp L, et al. Textural features in pre-treatment [F18]-FDG-PET/CT are correlated with risk of local recurrence and disease-specific survival in early stage NSCLC patients receiving primary stereotactic radiation therapy. Radiat Oncol [Internet]. BioMed Central Ltd.; 2015 [cited 2025 Jan 5];10:1–9. 10.1186/S13014-015-0407-7/TABLES/6. 10.1186/s13014-015-0407-7 PMC4465163 25900186

[46] Brose A, Miederer I, König J, Gkika E, Sahlmann J, Schimek-Jasch T, et al. Prognostic value of metabolic tumor volume on [18F]FDG PET/CT in addition to the TNM classification system of locally advanced non-small cell lung cancer. Cancer Imaging [Internet]. BioMed Central Ltd; 2024 [cited 2025 Jan 4];24:1–13. 10.1186/S40644-024-00811-7/FIGURES/5. 10.1186/s40644-024-00811-7 PMC11662478 39709461

[47] Jiménez-Sánchez J, Bosque JJ, Jiménez Londoño GA, Molina-García D, Martínez Á, Pérez-Beteta J, et al. Evolutionary dynamics at the tumor edge reveal metabolic imaging biomarkers. Proc Natl Acad Sci U S A [Internet]. National Academy of Sciences; 2021 [cited 2023 Oct 5];118:e2018110118. 10.1073/PNAS.2018110118/SUPPL_FILE/PNAS.2018110118.SAPP.PDF. 10.1073/pnas.2018110118 PMC8017959 33536339

[48] Tang X, Wu F, Chen X, Ye S, Ding Z. Current status and prospect of PET-related imaging radiomics in lung cancer. Front Oncol. Frontiers Media SA; 2023;13:1297674. 10.3389/FONC.2023.1297674/BIBTEX. 10.3389/fonc.2023.1297674 PMC10757959 38164195

[49] Kirienko M, Cozzi L, Antunovic L, Lozza L, Fogliata A, Voulaz E, et al. Prediction of disease-free survival by the PET/CT radiomic signature in non-small cell lung cancer patients undergoing surgery. Eur J Nucl Med Mol Imaging [Internet]. Springer Berlin Heidelberg; 2018 [cited 2025 Feb 3];45:207–17. 10.1007/S00259-017-3837-7/FIGURES/8 10.1007/s00259-017-3837-7 28944403

[50] Wang S, Belemlilga D, Lei Y, Ganti AKP, Lin C, Asif S, et al. Enhancing survival outcome predictions in metastatic non-small cell lung cancer through PET radiomics analysis. Cancers (Basel). 2024;16:3731. 10.3390/CANCERS16223731. 39594686 10.3390/cancers16223731 PMC11592397

[51] Pearl J. Graphical models for probabilistic and causal reasoning. Quantified Represent Uncertain Imprecision [Internet]. Dordrecht: Springer, Dordrecht; 1998 [cited 2025 Jan 4];367–89. 10.1007/978-94-017-1735-9_12.

[52] Hosny A, Aerts HJ, Mak RH. Handcrafted versus deep learning radiomics for prediction of cancer therapy response. Lancet Digit Health [Internet]. Elsevier Ltd; 2019 [cited 2023 Apr 13];1:e106–7. 10.1016/S2589-7500(19)30062-7. 10.1016/S2589-7500(19)30062-7 33323257

[53] Zhang X, Zhang Y, Zhang G, Qiu X, Tan W, Yin X, et al. Deep learning with radiomics for disease diagnosis and treatment: Challenges and potential. Front Oncol. Frontiers Media S.A.; 2022;12:276. 10.3389/FONC.2022.773840/BIBTEX. 10.3389/fonc.2022.773840 PMC8891653 35251962

[54] Hu Y, Jiang T, Wang H, Song J, Yang Z, Wang Y, et al. Ct-based subregional radiomics using hand-crafted and deep learning features for prediction of therapeutic response to anti-PD1 therapy in NSCLC. Physica Medica [Internet]. Elsevier; 2024 [cited 2025 May 8];117:103200. 10.1016/J.EJMP.2023.103200. 10.1016/j.ejmp.2023.103200 38160516

[55] Muhammad D, Bendechache M, Elsevier. Unveiling the black box: a systematic review of explainable artificial intelligence in medical image analysis. Comput Struct Biotechnol J [Internet]. 2024;24:542–60. 10.1016/J.CSBJ.2024.08.005. 39252818 10.1016/j.csbj.2024.08.005 PMC11382209

[56] Shi Y, Zou Y, Liu J, Wang Y, Chen Y, Sun F, et al. Ultrasound-based radiomics XGBoost model to assess the risk of central cervical lymph node metastasis in patients with papillary thyroid carcinoma: Individual application of SHAP. Front Oncol [Internet]. Frontiers Media S.A.; 2022 [cited 2025 Jun 28];12:897596. 10.3389/FONC.2022.897596/BIBTEX. 10.3389/fonc.2022.897596 PMC9458917 36091102

[57] Cheng X, Zhang Y, Zhu M, Sun R, Liu L, Li X. Predicting response to CCRT for esophageal squamous carcinoma by a radiomics-clinical SHAP model. BMC Med Imaging [Internet]. BioMed Central Ltd; 2023 [cited 2025 Jun 28];23:1–15. 10.1186/S12880-023-01089-0/FIGURES/4. 10.1186/s12880-023-01089-0 PMC10544369 37779188

[58] Retzlaff CO, Angerschmid A, Saranti A, Schneeberger D, Röttger R, Müller H, et al. Post-hoc vs ante-hoc explanations: xAI design guidelines for data scientists. Cogn Syst Res [Internet]. Elsevier; 2024 [cited 2025 Jun 28];86:101243. 10.1016/J.COGSYS.2024.101243.

[59] Ghassemi M, Naumann T, Schulam P, Beam AL, Chen IY, Ranganath R. A review of challenges and opportunities in machine learning for health. AMIA Summits on Translational Science Proceedings [Internet]. 2020 [cited 2025 Jun 28];2020:191. https://pmc.ncbi.nlm.nih.gov/articles/PMC7233077/. Accessed 28 Jun 2025. PMC7233077 32477638

[60] Chung HW, Lee KY, Kim HJ, Kim WS, So Y. FDG PET/CT metabolic tumor volume and total lesion glycolysis predict prognosis in patients with advanced lung adenocarcinoma. J Cancer Res Clin Oncol [Internet]. Springer; 2014 [cited 2025 Feb 3];140:89–98. 10.1007/S00432-013-1545-7/FIGURES/4. 10.1007/s00432-013-1545-7 PMC11823999 24194352

[61] Betancourt-Cuellar SL, Carter BW, Palacio D, Erasmus JJ. Pitfalls and limitations in non-small cell lung cancer staging. Semin Roentgenol. 2015;50:175–82. 10.1053/J.RO.2015.01.010. 26002237 10.1053/j.ro.2015.01.010

[62] Orlhac F, Frouin F, Nioche C, Ayache N, Buvat I. Validation of a method to compensate multicenter effects affecting CT radiomics [Internet]. Radiological Society of North America; 2019 [cited 2024 Sep 29];291:53–9. 10.1148/RADIOL.2019182023. 10.1148/radiol.2019182023 30694160

[63] Cruz-Jentoft AJ, Sayer AA, Elsevier. Sarcopenia. Lancet. 2019;393:2636–46. 10.1016/S0140-6736(19)31138-9. 31171417 10.1016/S0140-6736(19)31138-9

[64] Nardone V, Reginelli A, Grassi R, Boldrini L, Vacca G, D’Ippolito E, et al. Delta radiomics: a systematic review. Radiol Med. 2021;126:1571–83. 10.1007/S11547-021-01436-7. 34865190 10.1007/s11547-021-01436-7

[65] Cheng A, Hu G, Li WV. Benchmarking cell-type clustering methods for spatially resolved transcriptomics data. Brief Bioinform [Internet]. Oxford Academic; 2023 [cited 2024 Feb 18];24:1–12. 10.1093/BIB/BBAC475. 10.1093/bib/bbac475 PMC9851325 36410733

[66] Dong M, Wang L, Hu N, Rao Y, Wang Z, Zhang Y. Integration of multi-omics approaches in exploring intra-tumoral heterogeneity. Cancer Cell Int [Internet]. BioMed Central Ltd; 2025 [cited 2025 Sep 20];25:1–17. 10.1186/S12935-025-03944-2/FIGURES/5. 10.1186/s12935-025-03944-2 PMC12395700 40883767

[67] Goyette MA, Lipsyc-Sharf M, Polyak K. Clinical and translational relevance of intratumor heterogeneity. Trends Cancer. 2023;9:726–37. 10.1016/J.TRECAN.2023.05.001. 37248149 10.1016/j.trecan.2023.05.001 PMC10524913

[68] Yang J, Peng A, Wang B, Gusdon AM, Sun X, Jiang G, et al. The prognostic impact of lymph node metastasis in patients with non-small cell lung cancer and distant organ metastasis. Clin Exp Metastasis [Internet]. Springer Netherlands; 2019 [cited 2025 Oct 24];36:457–66. 10.1007/S10585-019-09985-Y/TABLES/6. 10.1007/s10585-019-09985-y 31420766

[69] Froelich JW, Salavati A. Artificial Intelligence in PET/CT Is about to make whole-body tumor burden measurements a clinical reality [Internet]. Radiological Society of North America ; 2019 [cited 2025 Oct 24];294:453–4. 10.1148/RADIOL.2019192425. 10.1148/radiol.2019192425 31825291

[70] Carvalho S, Leijenaar RTH, Troost EGC, Van Timmeren JE, Oberije C, Van Elmpt W, et al. 18F-fluorodeoxyglucose positron-emission tomography (FDG-PET)-Radiomics of metastatic lymph nodes and primary tumor in non-small cell lung cancer (NSCLC) – A prospective externally validated study. PLoS One [Internet]. Public Library of Science; 2018 [cited 2025 Oct 24];13:e0192859. 10.1371/JOURNAL.PONE.0192859. 10.1371/journal.pone.0192859 PMC5832210 29494598

