# Supplementary material for: An explainable imaging-clinical biomarker for non-small cell lung cancer prognostication based on normalised hotspot to centroid distance and [18F]FDG PET/CT radiomics
Source: Eur J Nucl Med Mol Imaging. 2025 Dec 12;53(5):3195–212. doi: 10.1007/s00259-025-07659-4 (PMC13013418; doi:10.1007/s00259-025-07659-4)
Supplement: Supplementary file 1 — Supplementary file1 (DOCX 1.09 MB) [file 259_2025_7659_MOESM1_ESM.docx]

**Supplementary Materials**

Intratumoural heterogeneity (ITH) is a well-acknowledged determinant of differential treatment responses among NSCLC patients and carries important prognostic value [1,2].

Given the retrospective observational design of our study, no biological data were available from patients in the external validation cohorts provided by our collaborating sites. With this limitation, we conducted an internally-validated, proof-of-concept study restricted to a subset of our discovery cohort (n = 64), focusing on cases with accessible histological features from their cancer tissue specimens. This included adenocarcinomas with described tumour growth patterns, based on a grading system adopted in literature [3].

**Integration of Histological Intratumoural Heterogeneity to Enhance Model Performance**

Biological indicators of intratumoural heterogeneity (ITH), spanning histological and molecular domains, are key determinants of differential treatment response and hold significant prognostic value. We performed this proof-of-concept sub-study, to investigate whether such indicators, in this case histologically observed tumour growth patterns, could improve predictive model performance,

**Method**

The discovery cohort (n = 64) for this sub-study was constructed from eligible cases from the main study discovery cohort. The eligibility criteria were 1) Adenocarcinomas, and 2) Available tumour histology based on hematoxylin and eosin (H&E) stained sections. The cohort was split into training and internal validation sets of 51:13 (80:20 ratio), respectively, balanced for patient’s age, tumour stage and prognosis; in keeping with the approach adopted in the main study.

Histologic evaluation was performed on H&E stained sections obtained from surgical resection specimens or diagnostic core biopsy samples. All slides were reviewed and reported by a consultant histopathologist with subspecialty expertise in lung cancer. Tumours were classified according to the 2015 World Health Organisation (WHO) criteria for lung adenocarcinoma [4], with the predominant, secondary, and tertiary architectural growth patterns annotated for each case, where appropriate. Growth patterns included lepidic, acinar, papillary, micropapillary, and solid subtypes.

To capture intratumoural histologic diversity, we derived simple ordinal heterogeneity scores from histopathological annotations: growth pattern heterogeneity was quantified based on the number and type of growth pattern (lepidic, acinar, papillary, micropapillary, solid). For each case, we enumerated the number of unique patterns present and assigned risk tiers according to established prognostic groups (0 = lepidic, 1 = acinar/papillary, 2 = micropapillary/solid). These were combined to generate a pattern-based intratumoural heterogeneity score (Histology-ITH), reflecting both the number and prognostic severity of patterns observed [3].

Histology-ITH was then combined with nLCEV using multivariable Cox regression on the training set to generate a composite signature, nLCEV-Histology-ITH; the performance of which was benchmarked to that of nLCEV in the internal validation set.

**Results**

Tumours with higher Histology-ITH scores, indicating greater histological diversity and presence of higher-risk growth patterns, were associated with poorer prognoses compared with tumours with lower scores (Fig. 10a). In a multivariable logistic model, both nLCEV and Histology-ITH are statistically significant predictors in a prognostic context (Fig. 10b). The composite nLCEV-Histology-ITH model achieved a performance superior to that of the nLCEV model confirming our hypothesis (Figs. 10c & 10d), although this was based on a small internal validation set (n = 13) and in the absence of external validation. These findings demonstrate the potential benefit of incorporating histological indicators of intratumoural heterogeneity into nLCEV for enhancing its performance.

**Discussion**

In this sub-study, histological features derived from H&E slides were used to generate a composite measure of intratumoural heterogeneity (Histology-ITH). By capturing both the diversity and prognostic severity of architectural growth patterns, this approach adds to the biological explainability of our biomarker, anchoring model outputs in well-established histopathological correlates of patient prognosis. Nonetheless, external validation could not be performed because histological data were unavailable in our independent testing cohorts; and more comprehensive characterisation of disease ITH was not possible due to the limited types of biological cancer data available.

An important drawback of using histology data as part of a predictive model is its reliance on invasive tissue sampling, usually achieved via core biopsy or surgical resection for NSCLC, with their procedural complications [5]. Liquid biopsy represents a promising alternative to capturing tumour-derived biological information [6]; however, it remains relatively experimental and carries its own limitations, such as lower sensitivity for spatial heterogeneity [7], challenges in standardisation [8], and the potential for sampling bias from circulating tumour material [9].

Future work includes the collection and integration of multi-modal biological indicators of ITH, such as genomic and molecular measurements (e.g. mutational burden, clonal diversity, copy number alterations, epigenetic variability) and microenvironmental factors (spatial variability in immune infiltration, stromal composition, hypoxia gradients, vascular irregularities). Incorporating these biological data into nLCEV could achieve predictive models with improved biological interpretability, performance and clinical utility.

**References**

1. Goyette MA, Lipsyc-Sharf M, Polyak K. Clinical and translational relevance of intratumor heterogeneity. Trends Cancer [Internet]. Cell Press; 2023 [cited 2025 Sep 22];9:726–37. <https://doi.org/10.1016/J.TRECAN.2023.05.001>
2. 2. Castello A, Russo C, Grizzi F, Qehajaj D, Lopci E. Prognostic Impact of Intratumoral Heterogeneity Based on Fractal Geometry Analysis in Operated NSCLC Patients. Mol Imaging Biol [Internet]. Mol Imaging Biol; 2019 [cited 2025 Sep 22];21:965–72. <https://doi.org/10.1007/S11307-018-1299-3>
3. Moreira AL, Ocampo PSS, Xia Y, Zhong H, Russell PA, Minami Y, et al. A Grading System for Invasive Pulmonary Adenocarcinoma: A Proposal From the International Association for the Study of Lung Cancer Pathology Committee. Journal of Thoracic Oncology [Internet]. Elsevier; 2020 [cited 2025 Sep 22];15:1599–610. <https://doi.org/10.1016/J.JTHO.2020.06.001>
4. Travis WD, Brambilla E, Nicholson AG, Yatabe Y, Austin JHM, Beasley MB, et al. The 2015 World Health Organization Classification of Lung Tumors: Impact of Genetic, Clinical and Radiologic Advances Since the 2004 Classification. Journal of Thoracic Oncology [Internet]. Elsevier; 2015 [cited 2025 Sep 23];10:1243–60. <https://doi.org/10.1097/JTO.0000000000000630>
5. Czyzewski A. ‘Virtual biopsy’ uses AI to help doctors assess lung cancer [Internet]. Imperial News. 2024 [cited 2024 Mar 3]. https://www.imperial.ac.uk/news/251593/virtual-biopsy-uses-ai-help-doctors/. Accessed 3 Mar 2024
6. Helman E, Nguyen M, Karlovich CA, Despain D, Choquette AK, Spira AI, et al. Cell-Free DNA Next-Generation Sequencing Prediction of Response and Resistance to Third-Generation EGFR Inhibitor. Clin Lung Cancer. Elsevier; 2018;19:518-530.e7. <https://doi.org/10.1016/J.CLLC.2018.07.008>
7. Cucchiara F, Petrini I, Romei C, Crucitta S, Lucchesi M, Valleggi S, et al. Combining liquid biopsy and radiomics for personalized treatment of lung cancer patients. State of the art and new perspectives. Pharmacol Res [Internet]. Pharmacol Res; 2021 [cited 2022 Nov 28];169. <https://doi.org/10.1016/J.PHRS.2021.105643>
8. Honoré N, Galot R, van Marcke C, Limaye N, Machiels JP. Liquid Biopsy to Detect Minimal Residual Disease: Methodology and Impact. Cancers (Basel) [Internet]. Cancers (Basel); 2021 [cited 2025 Sep 23];13. <https://doi.org/10.3390/CANCERS13215364>
9. Fairley JA, Cheetham MH, Patton SJ, Rouleau E, Denis M, Dequeker EMC, et al. Results of a worldwide external quality assessment of cfDNA testing in lung Cancer. BMC Cancer [Internet]. BioMed Central Ltd; 2022 [cited 2024 Dec 22];22:1–12. <https://doi.org/10.1186/s12885-022-09849-x>


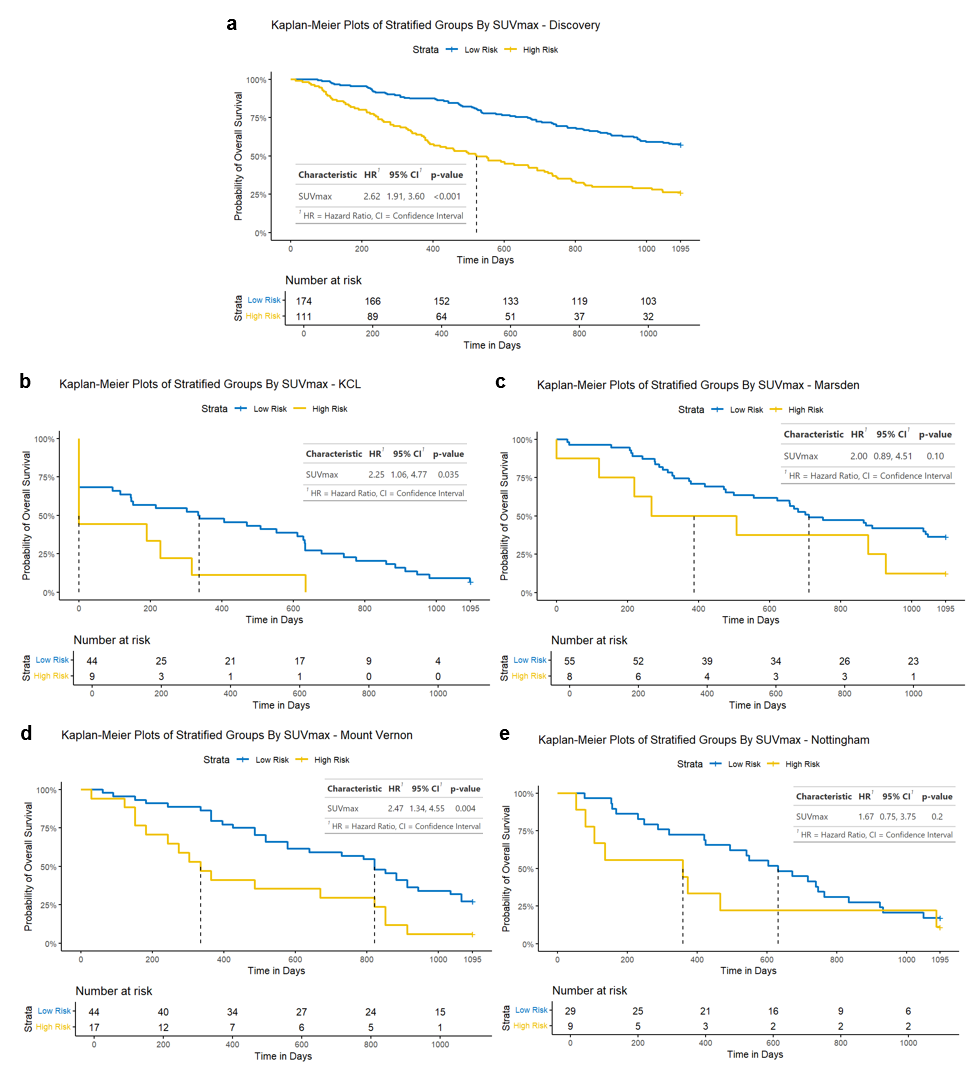


**Fig. 7** Performance of SUV_max_ for patient prognostication in the discovery and external validation cohorts: **a.** discovery, **b.** KCL, **c.** Marsden, **d.** Mount Vernon, and **e.** Nottingham. In addition to having HRs lower than that of nLCEV, SUV_max_ has not achieved statistically significant prognostic stratification (p<0.05) in the Marsden and Nottingham cohorts


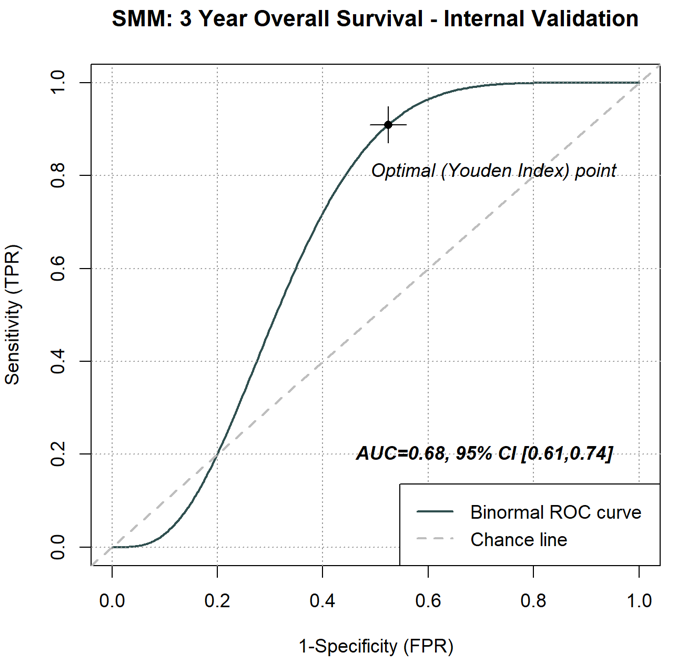


**Fig. 8** Receiver operating characteristics (ROC) analysis for evaluating the predictive performance for patients’ 3-year overall survival in the internal validation cohort by SMM. With an area under the curve (AUC) of 0.68 (95% CI [0.61, 0.74]); SMM was inferior to nLCEV’s AUC of 0.76 (95%CI [0.60, 0.92]).


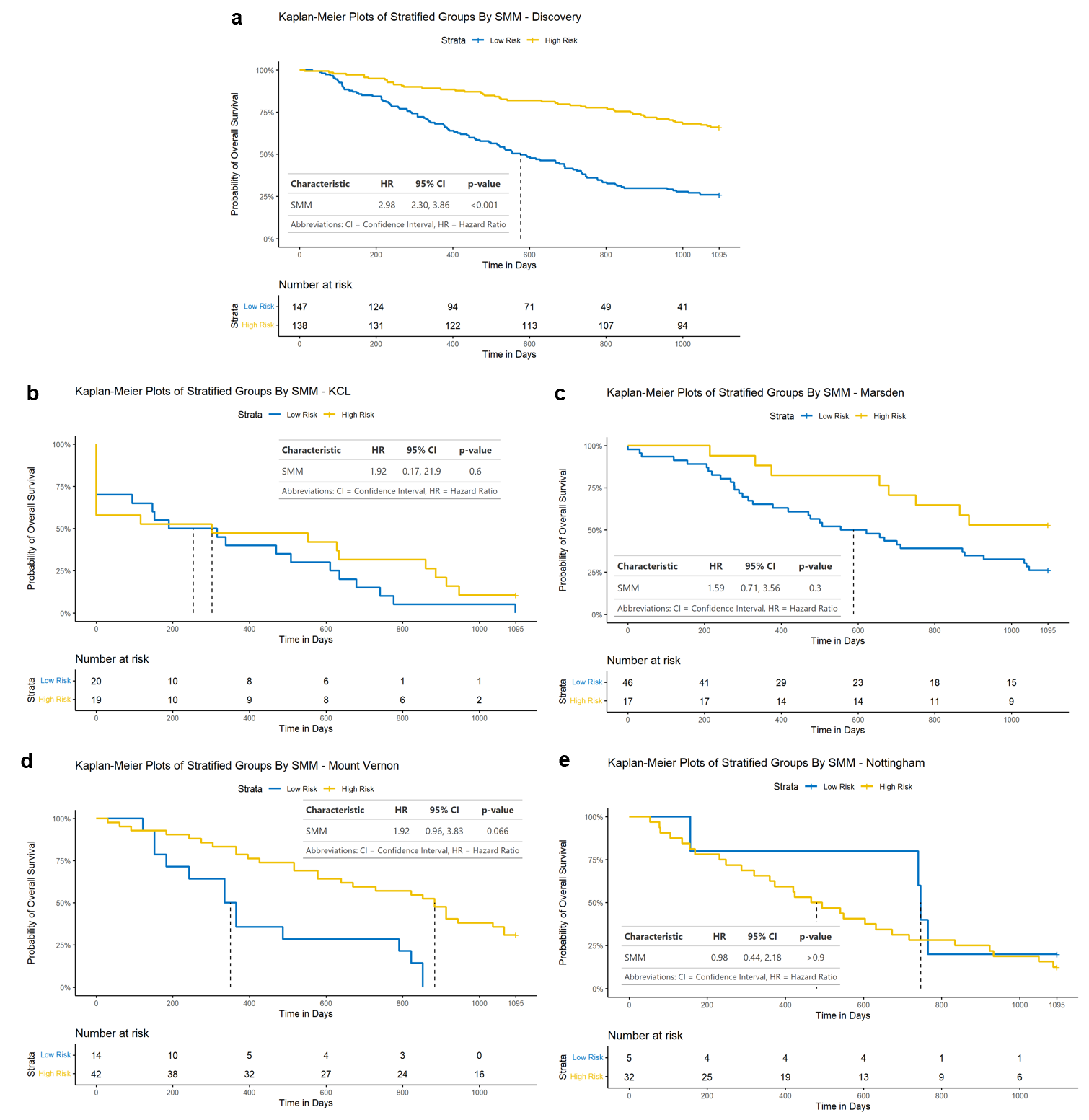


**Fig. 9** Performance of disease stage-metabolic model (SMM) for patient prognostication in the discovery and external validation cohorts: **a.** discovery, **b.** KCL, **c.** Marsden, **d.** Mount Vernon, and **e.** Nottingham. Whilst achieving effective prognostic stratification in discovery, it has failed to achieve similar performance in the external validation cohorts.


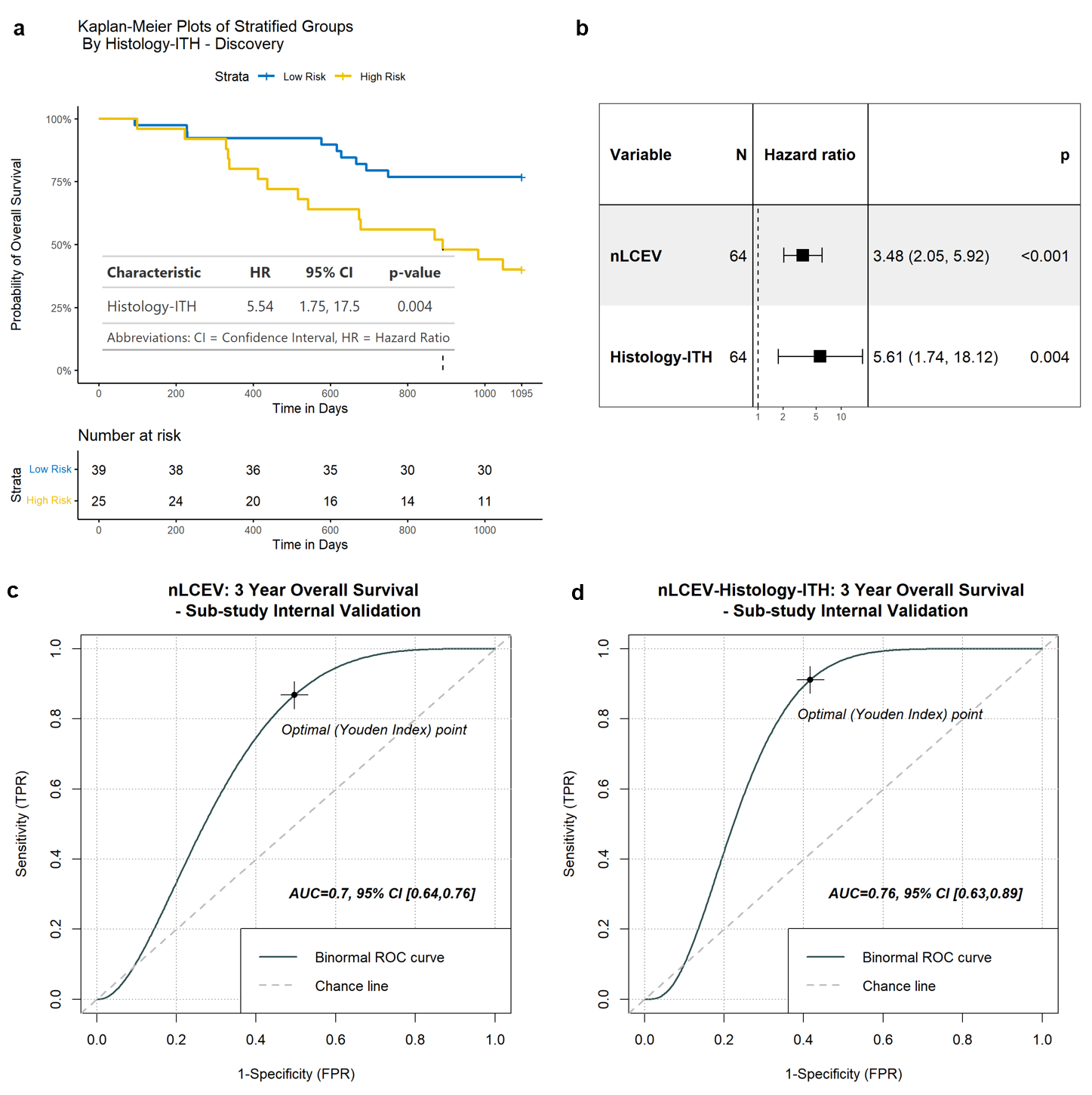


**Fig. 10 a.** Survival curve analysis of patients stratified based on their Histology-ITH score. **b.** Multivariable logistic regression of nLCEV and Histology-ITH showing the statistical significance of both metrics in a prognostic context. ROC analysis for 3-year OS prediction in the internal validation set by **c.** nLCEV, and **d.** nLCEV-Histology-ITH. Note in this data subset (n = 13), nLCEV-Histology-ITH achieved a superior performance to nLCEV.
